# Transcriptomic Analysis Reveals the Role of tmRNA on Biofilm Formation in *Bacillus subtilis*

**DOI:** 10.3390/microorganisms10071338

**Published:** 2022-07-01

**Authors:** Shanshan Xu, Qianqian Cao, Zengzhi Liu, Junpeng Chen, Peiguang Yan, Bingyu Li, Ying Xu

**Affiliations:** 1 Shenzhen Key Laboratory of Marine Bioresource and Eco-Environmental Science, Shenzhen Engineering Laboratory for Marine Algal Biotechnology, College of Life Sciences and Oceanography, Shenzhen University, Shenzhen 518055, China; xushanshan328@163.com (S.X.); caoqianqian520678@126.com (Q.C.); liuzz1990@outlook.com (Z.L.); 2060252001@email.szu.edu.cn (J.C.); 2 College of Physics and Optoelectronic Engineering, Shenzhen University, Shenzhen 518060, China; yanpg@szu.edu.cn; 3 Guangdong Key Laboratory for Genome Stability and Disease Prevention, Health Science Center, Shenzhen University, Shenzhen 518055, China

**Keywords:** tmRNA, biofilm formation, *Bacillus subtilis*, transcriptome, sporulation

## Abstract

*Bacillus* strains are widely distributed in terrestrial and marine environments, and some of them are used as biocontrol organisms for their biofilm-formation ability. In *Bacillus subtilis*, biofilm formation is fine-tuned by a complex network, a clear understanding of which still requires study. In bacteria, tmRNA, encoded by the *ssrA* gene, catalyzes *trans*-translation that can rescue ribosomes stalled on mRNA transcripts lacking a functional stop codon. tmRNA also affects physiological bioprocesses in some bacteria. In this study, we constructed a *ssrA* mutant in *B. subtilis* and found that the biofilm formation in the *ssrA* mutant was largely impaired. Moreover, we isolated a biofilm-formation suppressor of *ssrA*, in which the biofilm formation was restored to a level even stronger than that in the wild type. We further performed RNAseq assays with the wild type, *ssrA* mutant, and suppressor of *ssrA* for comparisons of their transcriptomes. By analyzing the transcriptomic data, we predicted the possible functions of some differentially expressed genes (DEGs) in the tmRNA regulation of biofilm formation in *B. subtilis*. Finally, we found that the overexpression of two DEGs, *acoA* and *yhjR*, could restore the biofilm formation in the *ssrA* mutant, indicating that AcoA and YhjR were immediate regulators involved in the tmRNA regulatory web controlling biofilm formation in *B. subtilis*. Our data can improve the knowledge about the molecular network involved in *Bacillus* biofilm formation and provide new targets for manipulation of *Bacillus* biofilms for future investigation.

## 1. Introduction

Biofilms are communities of surface-associated microbial cells enclosed by an extracellular matrix. Biofilms are the predominant mode of microbial growth in natural environments. Microbial biofilms are intimately related to human life. On the one hand, most human infections caused by microbial biofilms are notoriously challenging to treat because the microorganisms are embedded in a protective matrix of extracellular polymeric substances, for instance, exopolysaccharides (EPS) [1]. Apparently, the protection of biofilms confers multidrug resistance and immune evasion capabilities on bacteria, which makes them highly pathogenic and difficult to eradicate [2]. Biofilms formed by bacterial pathogens lead to prolonged infections and increased mortality rate among patients, which has always been a hot topic in medicine, in the past several decades [3]. On the other hand, biofilms produced by some bacteria can be beneficial to humans. They have shown great potential in wastewater treatment and production of microbial fuels [4,5]. The ability to form biofilms makes some *Bacillus* ideal biocontrol strains. For example, biofilms in *Bacillus subtilis* can protect plants from a variety of pathogens [6,7].

Given the importance of biofilm formation in *B. subtilis*, its molecular mechanisms have been extensively studied [8]. Biofilm formation in *B. subtilis* is finetuned by a complex network involving many regulatory factors. In general, the extracellular matrix of *B. subtilis* biofilms mainly includes extracellular polysaccharide (EPS), amyloid fiber (TasA) and the coat protein BslA, which are encoded by the *epsA-O* operon, *tapA-sipW-tasA* operon, and the *bslA* gene, respectively [9,10]. Regulatory factors control biofilm formation by directly or indirectly regulating the transcription of these matrix genes. To date, six crucial regulatory factors have been found to be involved in *B. subtilis* biofilm formation, namely Spo0A, AbrB, SinR, σ^H^, DegU, and CcpA [11,12]. Spo0A is central to the initiation of biofilm formation via protein phosphorylation [13,14]. The threshold level of Spo0A phosphorylation (Spo0A~P) can directly or indirectly inhibit the transcription of *abrB* and *sinR*, thereby diminishing the inhibition of *epsA-O* and *tapA-sipW-tasA* transcription by AbrB and SinR, and eventually promoting biofilm formation [15,16]. Recently, many other factors, such as Veg, YpqP, ComER, and ComX, have been described affecting biofilm formation in *B. subtilis* [17,18,19,20]. Despite the progres that have been made on the molecular mechanisms of *B. subtilis* biofilm formation, there are still unknown pathways underneath the known mechanisms, and a full understanding of the regulatory network requires more research.

During growth, ribosomes are stalled frequently on incomplete mRNA transcripts lacking a functional stop codon in bacterial cells. These result in nonstop complexes comprised of the stalled ribosome, incomplete mRNA, and nascent problematic polypeptide, the accumulation of which can cause the death of the cell [21]. Bacteria evolve mechanisms to recycle stalled ribosomes with trans-translation being the most utilized one. Trans-translation is mediated by transfer-messenger RNA (tmRNA) and SmpB. tmRNA possesses tRNA-like and mRNA-like structures with the mRNA domain coding for a tag peptide [22]. Briefly, to catalyze trans-translation, the alanine-charged tmRNA forms a complex with SmpB and EF-Tu·GTP, enters the vacant A site of the stalled ribosome and accepts the nascent peptide via transpeptidation. The ribosome then resumes moving by translation of the sequence encoded in the tmRNA, leading to the eventual release of the ribosome and addition of a C-terminal peptide tag to the incomplete nascent protein. The tagged polypeptide is recognized and degraded by various proteases, such as ClpXP and Lon [21]. Besides its biochemical function, tmRNA can affect bacteria physiologically. Some bacteria require tmRNA to be viable, such as *Shigella flexneri* and *Neisseria gonorrhoeae* [23,24]. tmRNA also regulates the stress response in *Escherichia coli*, pathogenesis in *Yersinia pseudotuberculosis*, and the cell cycle in *Caulobacter crescentus* [25,26,27]. A recent study suggests that the genome excision of P2 prophage negatively regulates biofilm formation, partially through trans-translation, in *Shewanella putrefaciens* [28]. In *B. subtilis*, it has been reported that under a variety of stressful conditions, such as high or low temperature, ethanol stress, and cadmium stress, the optimal growth of *B. subtilis* cells requires tmRNA [29,30]. In addition, it has been shown that in the *ssrA* (gene encoding tmRNA) mutant in *B. subtilis*, the activity of σ^K^, a factor playing a role in sporulation, is inhibited. Thus, tmRNA also affects sporulation of *B. subtilis* [31].

The connection between tmRNA and biofilms in *Bacillus* has not been proposed anywhere. Here, we used the CRISPER-Cas9 system to construct the *ssrA* mutant in the *B. subtilis* NCIB 3610 strain, and the results showed that the tmRNA mutant (TM) produced an obviously weaker biofilm than the wild-type strain (WT). Compared with WT, the swarming motility was also substantially hampered in TM. Moreover, a suppressor mutant strain tm3MB(MB) in the TM strain was isolated and showed an even stronger biofilm-formation phenotype than the WT strain. Finally, we analyzed the differentially expressed genes (DEGs) among WT, TM, and MB via RNAseq analysis. Among the DEGs, we found two genes, *acoA* and *yhjR*, that might encode putative immediate regulators of the pathway through which tmRNA affects biofilm formation in *B. subtilis* because overexpression of these genes partially or fully restored the biofilm-formation phenotype in TM. In this study, we found that tmRNA was required for biofilm formation and swarming motility in *B. subtilis*, which indicates that trans-translation is an important factor in the regulatory network of biofilm formation in this organism. We investigated the possible molecular mechanism how tmRNA affects biofilm formation, and our results shed a light on the understanding this complex biological process in *B. subtilis*.

## 2. Materials and Methods

### 2.1. Bacterial Strains and Growth Conditions

The *Bacillus* strains, *E. coli* strains and plasmids used in this study are listed in Table S1 in the Supplemental Material. Routine growth of *B. subtilis* and *E. coli* strains was performed in Luria–Bertani (LB) broth: 1% tryptone (Oxoid, Basingstoke, UK), 0.5% yeast extract (Oxoid) and 0.5% NaCl (Aladdin, Dubai, United Arab Emirates). The *B. subtilis* strains were grown in LBMG medium [32] (LB medium supplemented with MnCl_2_ and glycerol) for observation of pellicle biofilms. Colony biofilms were assayed in MSgg agar plates: 5 mM potassium phosphate, 100 mM MOPS [pH 7], 2 mM MgCl_2_, 50 μM MnCl_2_, 50 μM FeCl_3_, 700 μM CaCl_2_, 1 μM ZnCl_2_, 2 μM thiamine, 0.5% glycerol, 0.5% gutamate, and 50 μg/mL tryptophan and phenylalanine, as previously described [33]. Antibiotics were applied, if necessary, at the following concentrations: kanamycin, 50 μg ml^−1^ used for *E. coli* and 5 μg ml^−1^ used for *Bacillus* strains; chloramphenicol, 12.5 μg ml^−1^ used for *E. coli* and 5 μg ml^−1^ used for *Bacillus* strains; and streptomycin, 50 μg ml^−1^ used for *E. coli* strains.

### 2.2. Construction of Strains

To delete the *ssrA* gene in *B. subtilis* strains, we used the CRISPR-Cas9 system harbored in the plasmid vector pJOE8999, which is a shuttle plasmid containing a temperature-sensitive replicon for *B. subtilis* [34]. First, we subcloned the gRNA sequence for *ssrA* in pJOE8999 at the site of *Bsa* I, and obtained the resulting plasmid pYX101. Then, the DNA sequences located upstream and downstream of *ssrA* were PCR-amplified from the genomic DNA of strain NCIB 3610 using the primer pairs *ssrA*-U-F/R and *ssrA*-D-F/R, respectively. These two fragments were subsequently sutured together via SOE-PCR to construct the fragment *ssrA*-UD. The *ssrA*-UD fragment was subcloned in pYX101 at site Sfi I, and the resulting plasmid pYX102 was then transformed into JM110, to be demethylated as a new plasmid pYX103. Plasmid pYX103 was subsequently introduced into the target strain requiring a *ssrA* deletion via transformation, and the transformants were selected as Kan^R^ colonies. The verified Kan^R^ colonies were further grown at 45 °C to remove pYX103 that originated from the temperature-sensitive plasmid pJOE8999. The pYX103-free strains were screened as Kan^S^ colonies, and whether the disruption of *ssrA* via double-crossover homologous recombination occurred in these colonies during the incubation at 45 °C was tested using PCR with the primer pair *ssrA*-verify-F/R. Mutants with deleted genes *acoA*, *yhjR*, *sinR*, and *skfA* in *B. subtilis* were constructed in the same manner as that described for the *ssrA* mutant. To complement the *ssrA* mutation, plasmid pNW33N [35], a replicative and high-copy plasmid for *Bacillus*, was used as the vector carrying the wild-type ssrA gene. The sequence of *ssrA*, including its promoter region, was PCR-amplified from the genomic DNA of NCIB 3610 using the primer pairs *ssrA*-33N-F/R and obtained as a fragment flanked by the sequences of *Pst* I and *BamH* I. The amplified *ssrA* fragment was then subcloned in pNW33N between sites *Pst* I and *BamH* I, and the constructed plasmid was designated as pYX201 and transformed into JM110 for demethylation. The demethylated recombinant plasmid, pYX202, was finally transformed into the target host strains as described earlier [34]. In addition, to overexpress genes, we subcloned the fusion of a strong promoter pG and gene of interest in the pNW33N plasmid using the ClonExpress^®^ MultiS One Step Cloning Kit (Vazyme Biotech Co., Ltd., Nanjing, China), according to the manufacturer’s instructions. All the primers used in this study are listed in Table S2.

### 2.3. Biofilm Formation Assays

Strains were grown in LB to an optical density at 600 nm (OD_600_) of 1.0. For the colony assay, the LB cultures were 10 times diluted with sterile deionized water and spotted on the MSgg agar (1.5% agar), and the seeded agar plates were incubated at 30 °C for different times. For the pellicle assay, 1.5 μL of the diluted culture was added to 1.5 mL LBMG medium contained within a well of a 24-well microtiter plate, and the microtiter plates were incubated at 30 °C for the indicated times.

### 2.4. Swarming Motility Assay

To test swarming motility, single colonies of the strains grown on regular LB plates (1.5% agar) were picked and spotted onto the soft agar medium supplemented with LB broth and 0.65% agar. The seeded soft agar plates were incubated at 37 °C for 6 h, for observation of swarming zones.

### 2.5. Isolation of the Swarming Suppressors of ssrA in B. subtilis

The *ssrA* mutant was grown on regular LB plates and its single colonies were picked and spotted on the soft agar plates (the same as described above). The seeded plates were incubated at 37 °C till obvious swarming zones were observed. Bacteria from different areas of the outer rings of the swarming zones were picked and streaked on regular LB plates, for further purification of the suppressors of *ssrA*.

### 2.6. Confocal Laser Scanning Microscopy

The 48-h incubated submerged biofilms in microtiter plates were observed using a LSM710-Meta confocal laser scanning microscope (ZEISS, Jena, Germany). For the observation of biofilms in *B. subtilis* strains, cells were fluorescently stained in green with the nucleic acid marker SYTO^®^9 (Invitrogen by Thermo Fisher Scientific, Waltham, MA, USA), at the concentration of 5 μM (diluted in PBS). After a 20-min incubation in the dark at 37 °C to enable fluorescent labeling of the bacteria, the plate was mounted on the motorized stage of the confocal microscope. The biofilms were scanned at excitation wavelengths of 488 nm (argon laser; 3% intensity), with emission wavelengths collected from 493 to 550 nm for the SYTO^®^9 fluorescence. Three-dimensional (3D) projections of the biofilm structures were reconstructed using the Easy 3D function of the IMARIS software (Bitplane, Zürich, Switzerland) from the xyz image series.

### 2.7. Quantification of the Average Thickness and Biovolume of Biofilms

The average thickness and biovolumes of biofilms were calculated using COMSTAT [36]. The average biovolume was measured and calculated using results from three independent experiments, and the average thickness of biofilms was calculated using results from three independent experiments.

### 2.8. RNA Isolation

In order to select a suitable sampling time point, the growth conditions of WT, TM, and MB in the LBMG medium contained in 24-well microtiter plates were analyzed and the time point 36 h was selected. The cells at the selected time point were collected and their total RNA was extracted using the E.Z.N.A.^®^ Bacterial RNA Kit (OMEGA, Norcross, GA, USA), according to the manufacturer’s instructions. The RIN numbers and gel electropherogram of the RNA samples are shown in the Supplementary Materials.

### 2.9. Transcriptome Analysis

The library construction and Illumina sequencing were performed at Novogene Bioinformatics Technology Co., Ltd. (Beijing, China). A total amount of 3 μg RNA per sample was used as input material for the RNA sample preparations. mRNA was purified from total RNA using probes to remove rRNA. The purified mRNA was fragmented and used as templates for cDNA synthesis. The cDNA libraries were sequenced using Illumina HiSeq™ 2000. The genome of *B. subtilis* NCIB 3610 (NCBI Accession No. CP034484) was used as the reference for annotation. A total of 7,225,846 reads matched to the referenced genome in the sample of WT, 7,124,644 reads in the sample of TM, and 7,462,953 reads in the sample of MB. The differentially expressed genes (DEGs) were determined between WT and TM, TM and MB, and WT and MB with the standards of padj ≤0.05, and fold change |log_2_Ratio| > 1. The KEGG database (http://www.genome.jp/kegg/, accessed on 1 December 2020) was used to classify the genes with significant differential expression based on their functions. KOBAS software was used to test the statistical enrichment of differential expression genes in the KEGG pathways. In addition, we performed a Quantitative Real-time PCR experiment (Takara.RR430B TB Green Fast qPCR Mix) for the target genes to verify the reliability of the transcriptome data (see the Supplementary Materials).

### 2.10. Sporulation Assay

The sporulation assay was based on methods published in previous literature with some modifications according to the experimental requirements [37]. The samples were collected under the same conditions as described for the RNA isolation and serially diluted with sterile physiological saline. The collected cultures were well resuspended, centrifuged at 4500 rpm for 5 min, and then the supernatant was discarded. The harvested samples were washed with 500 μL physiological saline, and finally resuspended in physiological saline. The resuspended samples were subsequently subjected to mild sonication (20% amplitude, 1 s on, 1 s off for 5 s total) to liberate bacterial cells from the matrix. A 10 μL sub-sample of each cell sample was incubated for 20 min at 80 °C (heat-resistant) to kill vegetative cells. Then the samples were resuspended with 300 μL physiological saline, which was plated on LB agar plates and incubated for 12 h at 37 °C. The number of spores produced was determined as the number of colonies grown on the LB agar plates, for each sample. Meanwhile, 10 μL of each cell sample was directly resuspended with 300 μL physiological saline and were diluted (10,000 times) for plating. These samples were used as a control for the high-temperature treated samples.

### 2.11. Extraction of Extracellular Polysaccharide (EPS)

Colonies of the WT NCIB 3610 strain grown in the MSgg agar plates were crushed and transferred into a triangular flask. Sterile water was added to the flask for dissolution of the colonies, followed by incubation at 50 °C for 1.5–2 h, for extraction of the EPS. Then, the incubated mixture was centrifuged at 8000× *g* for 15 min, and the supernatant of the extract was collected. Next, 30% alcohol was added to the supernatant, which was subsequently precipitated at 4 °C for overnight. The supernatant was collected via centrifugation and further precipitated at 4 °C overnight, by adding 70% alcohol. The precipitate obtained was the extracted EPS. The precooled EPS samples were freeze-dried by a vacuum freeze-drying device for 24 h. The final white powdery substance was *Bacillus* crude EPS, which was weighed and quantified.

## 3. Results

### 3.1. tmRNA Positively Affects Biofilm Formation in B. subtilis

**Figure 1:**
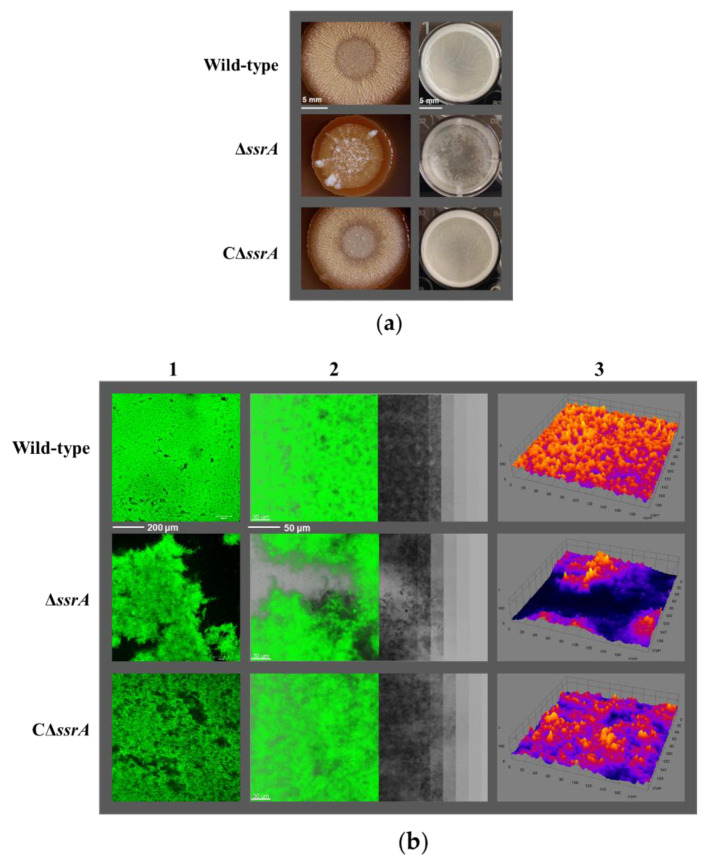

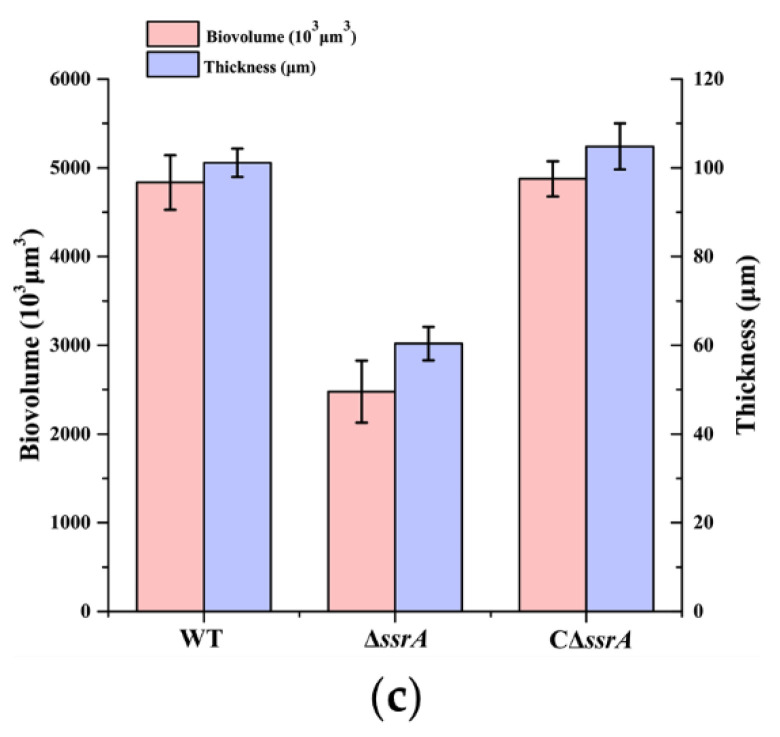
Comparison of the biofilm formation among WT, TM, and CΔ*ssrA*. (**a**) The WT, TM, and CΔ*ssrA* strains were grown in the MSgg agar plates for 3 days (left) and in 24-well microtiter plates supplied with the LBMG liquid medium for 48 h (right), and biofilms produced by the strains were observed. The images shown are the representatives of three independent experiments. The two scale bars both represent 5 mm. (**b**) Biofilms formed in the microtiter plates described in (A) were stained with STYO^®^9 and investigated using CLSM. Images of cells attached to the surface were shown in Lane 1. The confocal series were reconstructed through the software IMARIS and a representative image (left) with virtual shadow projection (right) is shown for each strain in Lane 2. The three-dimensional (3D) models of biofilms formed by these three strains are shown in Lane 3. The scale bars represent 200 μm and 50 μm, from left to right, respectively. (**c**) Quantification of the thickness and biovolume of the submerged biofilms produced by WT, TM, and CΔ*ssrA*. The biovolume (purple) and the thickness (pink) were calculated as average ± S.D. of six image series for each strain.

To study the effect of tmRNA on *B. subtilis* physiology, the *ssrA* deletion mutant in the type strain NCIB 3610 was constructed by using the CRISPR-Cas9 system (Figure S1). The biofilm formation in the wild-type (WT) and the *ssrA* mutant (TM) was investigated. The results showed that there was not a significant difference in biofilm formation between WT and TM when the strains were grown in MSgg medium (Figure S2a). However, in LB medium, TM produced obviously less pellicles than WT. The WT strain could form intact biofilms in LBM, while the TM strain could not (Figure S2a). When LBMG was used, the phenotype appeared to be stronger: WT started forming pellicles after 24-h incubation, whereas TM formed very little; WT formed more matured biofilms with wrinkles after 48-h incubation, whereas the TM only formed smooth or even broken biofilms at the same point (Figure S2a and Figure 1a). Thus, the LBMG medium was selected to grow bacteria for the following experiments required to be performed in liquid medium. The biofilm formation of WT and TM was also tested using different media plates. WT could produce biofilms with more wrinkled structures than TM during growth in all tested media plates (Figure S2b). Given that the MSgg medium agar plates produced the largest differences in biofilm-formation phenotypes, this medium was used to grow strains for observation of biofilms on solid surfaces (Figure 1a and Figure S2b).

To further confirm the effect of tmRNA on biofilm formation, the complementary strain (CΔ*ssrA*) was constructed and the biofilm-production defect was largely restored in CΔ*ssrA* (Figure 1a and Figure S2c,d). Moreover, the biofilm architectures were analyzed using Confocal Laser Scanning Microscope (CLSM). Focusing on the sheets of cells attached to the surface, there were obviously a larger number of cells observed for WT than that for TM (Figure 1b). The reconstructed images and 3D models of the confocal series showed that the biofilms produced by TM were much weaker and contained less protruded structures than those produced by WT (Figure 1b). The phenotypes in the surface-attached cells and biofilm formation observed for CΔ*ssrA* resembled those of WT (Figure 1b). Furthermore, the thickness and biovolume of biofilms formed by the strains described above was quantified. The average thicknesses of biofilms produced by WT, TM, and CΔ*ssrA* were 101 μm, 60 μm, and 104 μm, respectively. The average biovolume calculated for WT, TM, and CΔ*ssrA* were 4.834 × 10^3^ μm^3^, 2.478 × 10^3^ μm^3^, and 4.875 × 10^3^ μm^3^, respectively (Figure 1c). All these results suggest that tmRNA plays an important role in biofilm initiation and maturation in *B. subtilis*.

### 3.2. Isolation of a Suppressor of ssrA That Cured the TM Biofilm-Formation Defect

**Figure 2:**
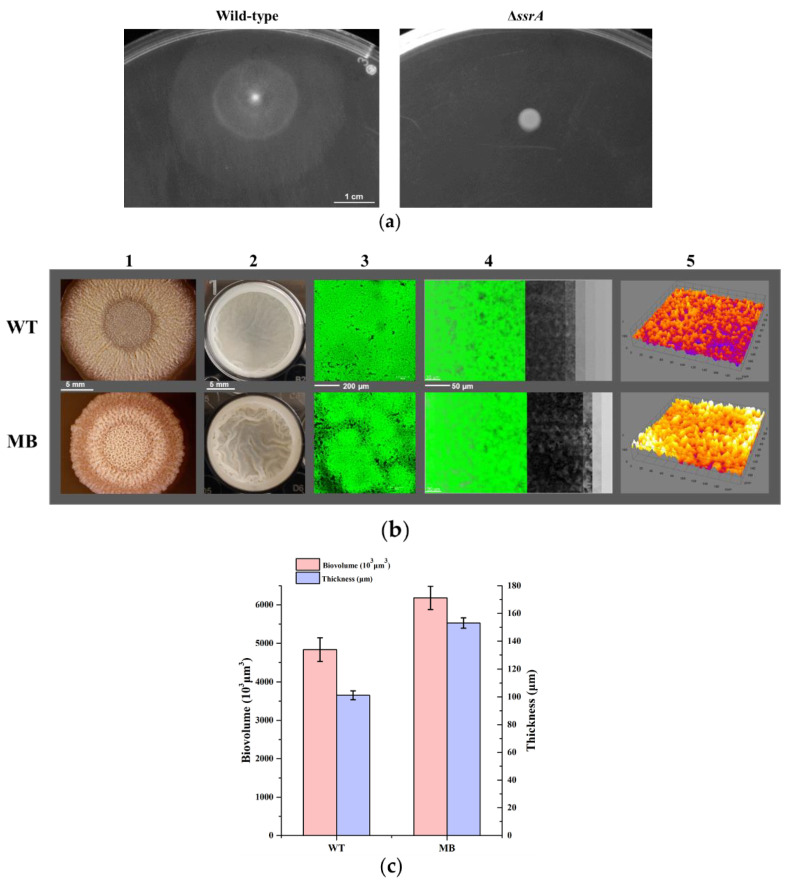
Screening for the suppressor(s) of *ssrA* that possesses restored biofilm formation ability. (**a**) To test the swarming motility, WT and TM were grown in soft agar plates (0.65% agar) for 6 h at 37 °C. The scale bar represents 1 cm. The images shown are the representatives of three independent experiments. (**b**) The WT and MB strains were grown in the MSgg agar plates for 3 days (Lane 1) and in 24-well microtiter plates supplied with the LBMG liquid medium for 48 h (Lane 2), and biofilms produced by the strains were observed. The images shown in Lane 1 and Lane 2 are the representatives of three independent experiments. Biofilms formed in the microtiter plates were stained with SYTO^®^9 and investigated using CLSM. Images of cells attached to the surface were shown in Lane 3. The confocal series were reconstructed through the software IMARIS and a representative image (left) with virtual shadow projection (right) is shown for each strain in Lane 4. The 3D models of biofilms formed by these two strains are shown in Lane 5. The scale bars in Lane 1–4 represent 5 mm, 5 mm, 200 μm, and 50 μm, respectively. (**c**) Quantification of the thickness and biovolume of the submerged biofilms produced by WT and MB. The biovolume (purple) and the average thickness (pink) were calculated as the average and ±S.D. of a six-image series for each strain.

In view of the association of biofilm formation with swarming motility in *B. subtilis* [38], in this study, the swarming motility in colonies of the WT strain showed obvious swarming zones, and yet colonies of the TM strain showed almost no swarming zones (Figure 2a). This result indicates that lacking tmRNA caused dramatically diminished swarming motility in *B. subtilis*. It is likely that certain factors involved in the tmRNA regulation of biofilm formation also affect swarming motility in *B. subtilis*.

It has been described that swarming suppressor mutants for low-motile *B. subtilis* strains can be isolated via prolonged incubation of the bacteria on soft agar plates [39]. Here, one of the strains obtained (designated as tm3MB (MB)) had a not only restored but reinforced biofilm-formation phenotype (Figure 2b and Figure S3). The biofilms of MB were much thicker than those of WT, the average thickness of biofilms was 153 μm (MB) and 101 μm (WT) and the average biovolume calculated was 6.185 × 10^3^ μm^3^ (MB) and 4.834 × 10^3^ μm^3^ (WT) (Figure 2b,c). All these results indicated that the suppressor mutant (MB) cured the TM biofilm-formation defect. However, what mutations led to the enhanced biofilm formation in MB and are there novel unknown factors involved in biofilm formation in *B. subtilis*? To answer these questions, more in-depth experiments are required.

### 3.3. Transcriptome Analysis among the WT, TM, and MB Strain

**Figure 3:**
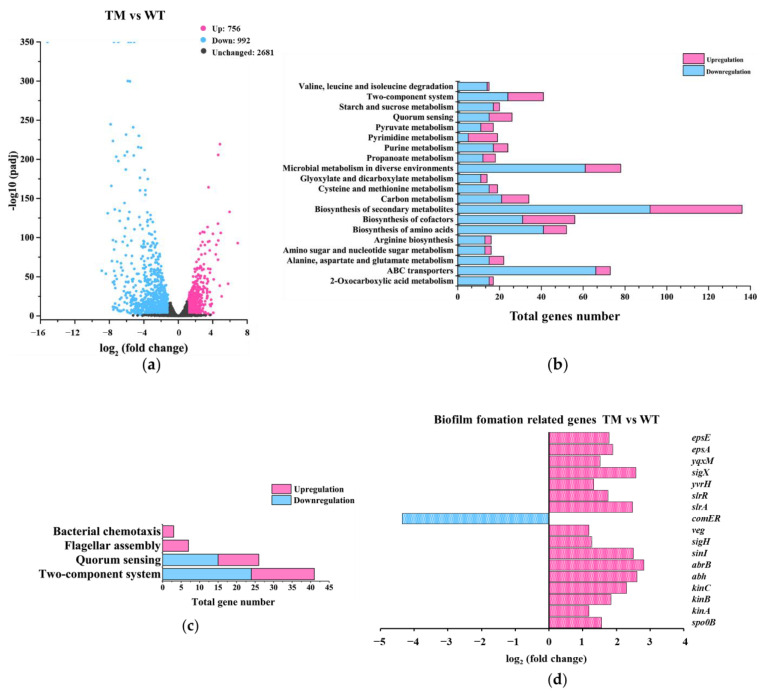
Analysis of the gene-expression differences between TM and WT. (**a**) A volcano plot showing the DEGs found in TM compared with WT. DEGs were defined as the genes whose expression was up-regulated (pink dots) or down-regulated (blue dots) with a fold change of two or more (*p* ≤ 0.05), when TM was compared with WT. Black dots represent genes without a significant change in expression. (**b**) Profiling of the DEGs via using the annotation of KEGG. (**c**) Grouping the DEGs by the indicated metabolic pathways. (**d**) Changes in the expression of the known biofilm−formation genes found as DEGs.

**Figure 4:**
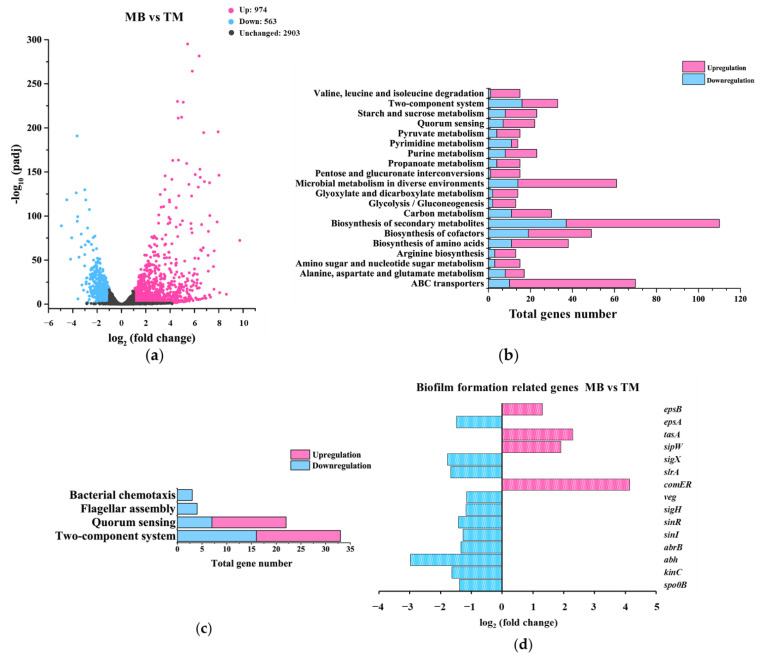
Analysis of the gene-expression differences between TM and MB. (**a**) A volcano plot showing the DEGs found in MB, compared with TM. DEGs were defined as the genes whose expression was up-regulated (pink dots) or down-regulated (blue dots) with a fold change of two or more (*p* ≤ 0.05), when MB was compared with TM. Black dots represent genes without a significant change in expression. (**b**) Profiling of the DEGs via using the annotation of KEGG. (**c**) Grouping the DEGs by the indicated metabolic pathways. (**d**) Changes in the expression of the known biofilm-formation genes found as DEGs.

To investigate whether the tmRNA mutation and suppressor mutation(s) in MB caused any altered gene expression in the parent strain, an RNAseq analysis of WT, TM, and MB was performed. The quality of the RNA samples is shown in Figure S4. The results show that nearly 40% of the genes across the genome were differentially expressed in TM, compared with WT (Figure 3a), suggesting a major role of tmRNA in genetic regulation in *B. subtilis*. Furthermore, compared with TM, there are 1537 genes which had altered expression in MB (Figure 4a), indicating that the spontaneous suppressor mutation(s) in MB resulted in great reprogramming of gene expression in TM. Out of these 1537 genes, there are 1234 whose expression was also altered when comparing TM with WT, suggesting that most of the gene-expression differences found in MB were tmRNA related (Figure S5). In addition, 178 genes were found differentially expressed in all the three comparisons (Figure S5). Further study of the differentially expressed genes (DEGs) found in TM and MB might provide us valuable information regarding the effect of tmRNA on *B. subtilis* physiology, especially biofilm formation.

#### 3.3.1. Gene-Expression Differences between TM and WT

There were 756 genes up-regulated and 992 genes down-regulated with a fold change of two or more comparing TM with WT (Figure 3a). These genes were classified into 20 major cellular processes based on the annotation of KEGG (Figure 3b), and further grouped into three major metabolic pathways related to biofilm formation, including chemotaxis, flagellar assembly and quorum sensing (Figure 3c). Overall, the results showed that tmRNA mediated the expression of genes involved in many cellular processes with biosynthesis of secondary metabolites, microbial metabolism in diverse environments, and ABC transporters being the most mediated ones (Figure 3b). Surprisingly, the expression of known genes required for chemotaxis and flagellar assembly was either not altered or even increased in TM (Figure 3c), indicating that tmRNA affected *B. subtilis* swarming motility and biofilm formation without repressing the transcription of flagellar genes or through flagellar-independent mechanism(s). Moreover, tmRNA could either positively or negatively regulate the expression of some genes involved in quorum sensing (Figure 3b,c). According to the DEGs, tmRNA regulated the transcription of genes encoding for known factors controlling biofilm formation in a highly unusual way (Figure 3d). For instance, tmRNA could not only repress the transcription of some known activators, such as SigX, Abh, EpsA, and EpsE, but also some repressors, such as AbrB, of *B. subtilis* biofilm formation (Figure 3d). In addition, tmRNA was required for the expression of *comER*, the product of which is an activator for both biofilm formation and sporulation in *B. subtilis* (Figure 3d). All these data suggest that tmRNA functions as an important regulator controlling many cellular processes in *B. subtills*, possibly via a highly complex network.

Among these DEGs, the genes whose expression was greatly affected (top 20) by tmRNA were analyzed (Tables S3 and S4). The genes with an expression pattern that was either consistent with or opposite to the pattern of biofilm formation in WT, TM, and MB were focused on. For instance, the expression of gene *ytcB* and *acoA* was decreased in TM and yet elevated in MB, compared with the WT (Table S4), which was regulated in a similar manner as that found for biofilm formation. These data suggested that AcoA and YtcB are potential intermediate activators within the tmRNA regulatory cascade(s) of biofilm formation in *B. subtilis*. It has been described that during biofilm development, the acetoin utilization genes (*acoABCL* operon) expression was among the most significant upregulation [40]. In addition, *acoA* and *acoB* can be coregulated by interconnected webs of transcriptional factors, including Fnr, AcoR, SigL, and CcpA [41]. On the other hand, the product of ytcB is a putative UDP-glucose epimerase, which is expressed late during sporulation in the mother cell, and its expression can be inhibited by GerE and σ^K^ [42]. These results indicated the tmRNA has effects on acetoin metabolism and UDP-glucose modification, the study of which might provide important information elucidating the mechanism(s) for the tmRNA regulation of biofilm formation in *B. subtilis*.

#### 3.3.2. Gene-Expression Differences between MB and TM

Comparing MB with TM, there were 974 genes up-regulated whereas 563 genes were down-regulated with a fold change of two or more (Figure 4a). In addition, these genes were classified into 20 major cellular processes based on the annotation of KEGG (Figure 4b), and further grouped into three major metabolic pathways related to biofilm formation (Figure 4c). These data showed that compared with TM, the most affected cellular processes in MB were the biosynthesis of secondary metabolites, microbial metabolism in diverse environments, and ABC transporters, which was the same as that found in the comparison between TM and WT (Figure 4b). In addition, the distribution of up-regulated and down-regulated genes in the shown bioprocesses comparing MB with TM was almost opposite to that in the TM with WT comparison (Figure 3b and Figure 4b), suggesting that the gene-expression difference caused by tmRNA could be largely restored via the suppressor mutation(s) in the MB strain. Still, the DEGs involved in metabolisms of chemotaxis, flagellar biosynthesis, quorum sensing, and biofilm formation did not provide us sufficient information to unveil the mechanisms underneath the physiological phenotypes found in MB and TM (Figure 4c,d).

We also analyzed the most up-regulated genes (Table S5) and down-regulated genes (Table S6) in the MB with TM comparison. The data showed that all the genes up-regulated in TM, compared with WT, were down-regulated in MB, compared with TM, and vice versa (Tables S5 and S6). This result further confirmed the tight connection between tmRNA and the suppressor mutation(s) in MB. Moreover, the genes of *acoA*, *ytcB*, *acoL*, *gerPA*, *yngJ*, and *ydgA* were down-regulated in TM and yet overexpressed in MB, compared with WT, the expression pattern of which resembled the biofilm-formation pattern observed in these three strains (Table S5). In contrast, *hxlB* and *rocA* were expressed totally in a reverse manner, in WT, TM, and MB (Table S6). Among these genes, *acoA* and *ytcB* have been described above, and *acoL* belongs to the *acoABCL* operon. *gerPA* encodes a germination protein GerPA, and this gene is expressed during sporulation in the mother cell. Expression of the σ^K^-dependent *gerP* operon is repressed by GerE [43,44]. The yngJ gene is located in the *yngJIHGFE* gene cluster in *B. subtilis*, which is expressed during sporulation in the mother cell. According to the profiling based on Swiss-Prot, YngJ is a homologue of acyl-CoA dehydrogenase [45]. The *ydgA* gene encodes a conserved hypothetical protein, and the expression of this gene is regulated by σ^K^ [46]. In addition to AcoA and YtcB, AcoL, GerPA, YngJ, and YdgA could be potential activators of biofilm formation, involved in the regulatory cascade(s) of tmRNA. On the other hand, gene products of *hxlB* and *rocA* are also noteworthy because they could be potential repressors of biofilm formation within the tmRNA regulatory web. HxlB is a 6-phospho-3-hexuloisomerase involved in the ribulose monophosphate pathway in *B. subtilis* [47]. RocA is a L-glutamate gamma-semialdehyde dehydrogenase involved in proline metabolism [48]. Clearly, all these results provided us more information about how tmRNA might affect *B. subtilis* physiology.

#### 3.3.3. Gene-Expression Differences between MB and WT

**Figure 5:**
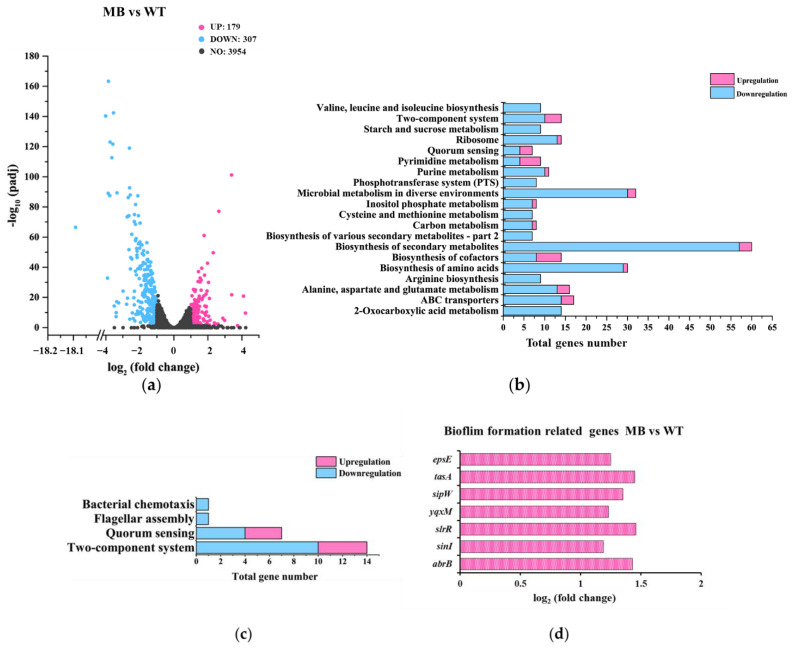
Analysis of the gene-expression differences between MB and WT. (**a**) A volcano plot showing the DEGs found in MB, compared with WT. DEGs were defined as the genes whose expression was up-regulated (pink dots) or down−regulated (blue dots) with a fold change of two or more (*p* ≤ 0.05), when MB was compared with WT. Black dots represent genes without a significant change in expression. (**b**) Profiling of the DEGs via using the annotation of KEGG. (**c**) Grouping the DEGs by the indicated metabolic pathways. (**d**) Changes in the expression of the known biofilm-formation genes found as DEGs.

Comparing MB with WT, there were 179 genes up-regulated and 307 genes down-regulated with a fold change of two or more (Figure 5a). These genes were classified into 20 major cellular processes based on the annotation of KEGG (Figure 5b). Clearly, the number of DEGs found in this comparison was much smaller than that found in the other two comparisons described above (Figure 3a and Figure 4a). This might be because most of the gene-expression differences resulted from the tmRNA deletion were restored to a WT level in the MB strain. In fact, more than half of the DEGs shown in Figure 5 were also found in the comparison between TM and WT. Thus, the profiling results for the DEGs in the MB vs. WT comparison were similar to those found in the TM vs. WT comparison (Figure 3b and Figure 5b). Notably, there are bioprocesses, such as ribosome metabolism, which seemed to be uniquely caused by the suppressor mutation(s) in MB rather than the tmRNA deletion (Figure 5b). Grouping the DEGs in the MB vs. WT comparison into biofilm-related metabolic pathways did not provide sufficiently precise information (Figure 5c). Yet, profiling of these DEGs based on known biofilm-formation genes showed that, in addition to *tapA*, the other two genes *sipW* and *tasA* included in the same operon were up-regulated in MB, which was not observed in the TM vs. WT comparison. Further study of this operon might provide information elucidating the mechanism of the over-produced biofilms in MB (Figure 2).

Again, we analyzed the most up-regulated genes (Table S7) and down-regulated genes (Table S8) in MB, compared with WT. We still focused on the genes with an expression pattern that was similar to *acoA* or *hxlB*, as described in the comparison between TM and MB. Two more genes, *yhjR* and *yxbB*, possessed an *acoA*-similar expression pattern in strains of WT, TM, and MB. The gene *yhjR* encodes a spore coat protein with the function of protecting the spores, and its localization depends on SafA [49]. The *yxbB* gene encodes a putative S-adenosyl-L-methionine-dependent methyltransferase, whose function has not been studied, according to the information based on the SubtiWiki database [50]. Gene *yxbB* is located in the *asnH* operon comprising *yxbB*, *yxbA*, *yxnB*, *asnH*, and *yxaM*, which is induced dramatically during the transition from exponential to stationary phase when the cells are grown in rich sporulation medium [51]. These data suggest that YhjR and YxbB might also be intermediates of the tmRNA regulatory pathway(s) for biofilm formation in *B. subtilis*.

### 3.4. Elucidating the Roles of Known Biofilm-Formation Factors in the tmRNA Regulation of Biofilm in B. subtilis

**Figure 6:**
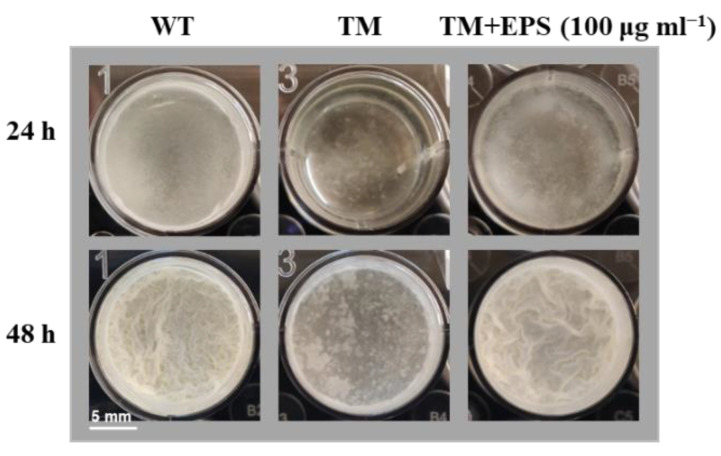
The effect of EPS on TM biofilm formation. The strains of WT and TM were grown in the LBMG liquid medium, supplemented with or without 100 μg mL^−1^ EPS, and observed for biofilm formation. The scale represents 5 mm.

Given that the extracellular matrix is an important structure for biofilm complexes, we extracted the EPS from WT, and added the obtained EPS into TM cultures. As shown in Figure 6, the biofilm-formation ability of TM was restored almost to the WT level, when 100 μg mL^−1^ EPS was added to the cultures. This result indicates that tmRNA affects biofilm formation through positively regulating EPS production in *B. subtilis*. The transcriptome results regarding EPS production were then checked precisely. Unfortunately, as described earlier for Figure 3d, the known EPS-related genes were regulated in an unclear manner. For instance, both the structure genes, such as *epsA* and *epsE*, and the repressor gene *abrB* were up-regulated in TM (Figure 3d and Table S9), which appeared to be paradoxical and not consistent with the biofilm-formation phenotypes we found here. In MB, the *epsA* expression was restored to a WT level, whereas the *epsE* expression was not altered significantly, and the *epsB* expression was increased obviously, compared with TM (Table S9), which contributed nothing to explain the tmRNA effect on EPS production. Moreover, some other extracellular-matrix activator genes such as *tapA*, *sigX*, and *abh* were also up-regulated in TM (Figure 3d and Table S9), making the tmRNA regulatory network more complex. Interestingly, the whole operon of *tapA-sipW-tasA* was overexpressed in MB, compared with WT, which was not observed when comparing TM with WT (Figure 5d and Table S9). Given that the *tapA-sipW-tasA* operon encodes the amyloid fiber of the extracellular matrix, it is worth investigating whether the over-produced biofilms were (partially) related to the overexpression of this operon in MB. However, this phenotype specifically found in MB is likely to be tmRNA-independent. All these data suggested that tmRNA might affect the known EPS-related genes at post-transcriptional levels or it controlled unknown pathway(s) producing the extracellular matrix in *B. subtilis*.

It is known that SinR represses the expression of matrix genes in *B. subtilis* [52]. Here, the transcriptome results show that, compared with WT, expression of *sinR* was increased by approximately 50% in TM and decreased by approximately 45% in MB. which was consistent with the biofilm-formation pattern in these three strains. Subsequently, we deleted the *sinR* gene in TM to test whether the biofilm-formation phenotype could be restored. Unfortunately, our results show that the deletion of *sinR* did not rescue the biofilm defect in TM (Figure S6), suggesting the existence of other pathways involved in the tmRNA regulation of biofilm formation in *B. subtilis*.

### 3.5. Initial Characterization of the Unknown Pathways Involved in the tmRNA Regulation of Biofilm Formation in B. subtilis

**Figure 7:**
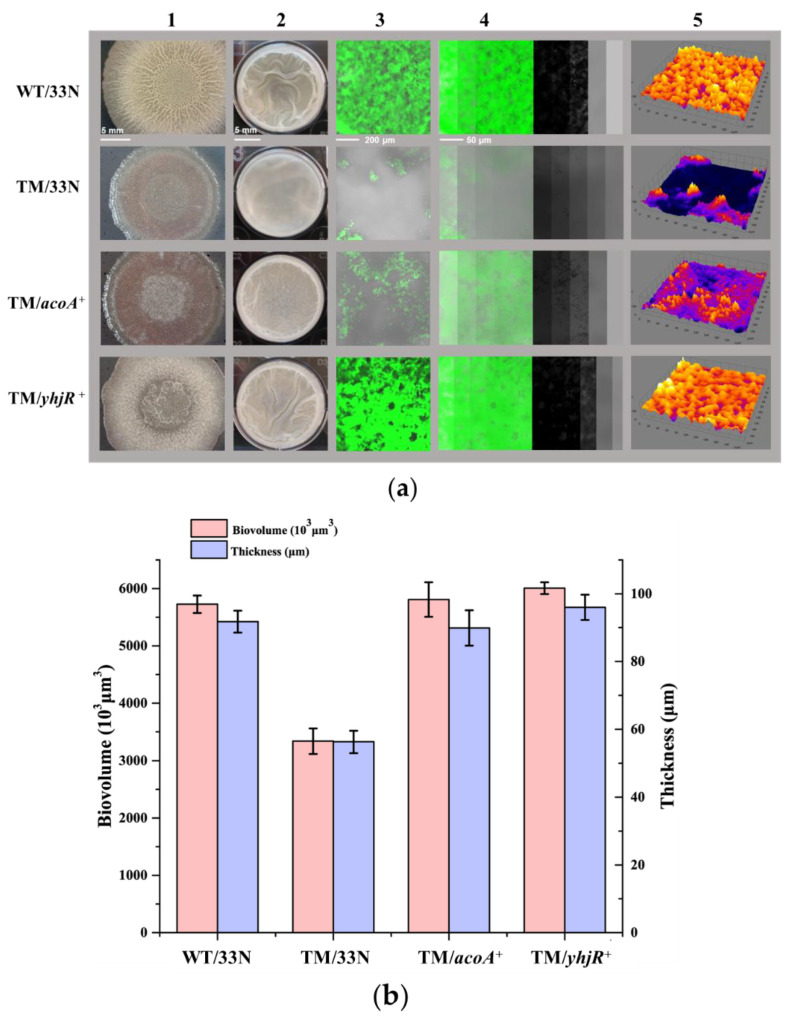
Comparison of the biofilm formation among WT/33N, TM/33N, TM/*acoA*^+^, and TM/*yhjR*^+^. (**a**) The strains of WT/33N, TM/33N, TM/*acoA*^+^, and TM/*yhjR*^+^ were grown on the MSgg agar plates for 3 days (Lane 1) and in 24-well microtiter plates supplied with the LBMG liquid medium for 48 h (Lane 2), and biofilms produced by the strains were observed. The images shown in Lane 1 and Lane 2 are the representatives of three independent experiments. Biofilms formed in the microtiter plates were stained with STYO^®^9 and investigated using CLSM. Images of cells attached to the surface are shown in Lane 3. The confocal series were reconstructed through the software IMARIS and a representative image (left) with virtual shadow projection (right) is shown for each strain in Lane 4. The 3D models of biofilms formed by these two strains are shown in Lane 5. The scale bars in Lane 1–4 represent 5 mm, 5 mm, 200 μm, and 50 μm, respectively. (**b**) Quantification of the thickness and biovolume of the submerged biofilms produced by these four strains. The biovolume (purple) and the thickness (pink) were calculated as the average and ±S.D. of a serious of six images for each strain.

To better understand how tmRNA functions in the complex regulatory network for biofilm formation in *B. subtilis*, the effects of several genes (*acoA*, *acoL*, *gerPA*, *ydgA*, *yhjR*, *yngJ*, *ytcB*, and *yxbB*) whose transcription was highly regulated by tmRNA were investigated. Here, all these genes are over-expressed in TM, but only overexpression of *acoA* and *yhjR* could cause restored phenotypes in biofilm formation in the TM strain (Figure 7a and Figure S7).

As shown in Figure 7a, the strain of TM/*acoA*^+^ possessed stronger biofilm formation in liquid cultures, and this phenotype was not obvious on agar plates. However, biofilms produced by the TM/*acoA*^+^ strain were still weaker than WT. The reconstruction of confocal images showed that the overexpressed *acoA* enabled TM to produce intact biofilms rather than broken biofilms (Figure 7a). In addition, the thickness and biomass of the TM/acoA^+^ pellicles were similar to that of the WT pellicles (Figure 7b). These data indicate that the overexpression of *acoA* could partially restore the biofilm formation in TM. To further verify the role of AcoA in biofilm formation, the *acoA* gene was deleted in WT and MB. It is found that when grown in the liquid medium, the Δ*acoA* mutants in both WT and MB showed weaker biofilm production than the parent strains, after incubation for 24 h. However, this phenotype was not observed for the Δ*acoA* mutants after incubation for 48 h (Figure S8a). These results suggest that the Δ*acoA* mutation could cause delayed biofilm formation in *B. subtilis* grown in the LBMG liquid medium. No obvious phenotypes in biofilm formation were found when the strains were grown on the MSgg agar plates (Figure S8b).

The overexpression of *yhjR* appeared to restore the biofilm production in TM more efficiently. As shown in Figure 7a, on the MSgg agar plate, the recovery of the biofilm formation in TM/*yhjR*^+^ was quite obvious, and in the LBMG liquid medium, the pellicles formed by strain TM/*yhjR*^+^ were comparable to those of the WT strain. The reconstruction of confocal images showed that the *yhjR* overexpression enabled TM to fully restore the biofilm formation (Figure 7a), and the thickness and biomass of the TM/*yhjR*^+^ pellicles were similar to that of the WT pellicles (Figure 7b). These data indicate that the overexpression of *yhjR* could largely complement the biofilm formation in TM. However, we failed to describe the biofilm-formation phenotypes in the *yhjR* mutants because different isolates, which were supposed to be genetically identical, of the *yhjR* mutant in WT showed various phenotypes in biofilm formation. More experiments are required to explain and resolve this problem in our future studies. In summary, all these data suggest that the tmRNA regulation of biofilm might involve pathways of acetoin metabolism (AcoA) and sporulation (YhjR) in *B. subtilis*. More experiments in the future are needed to explain the AcoA and YhjR functions in the same or independent regulatory cascade originated from tmRNA.

### 3.6. Sporulation in WT, TM, and TM Derivates

**Figure 8:**
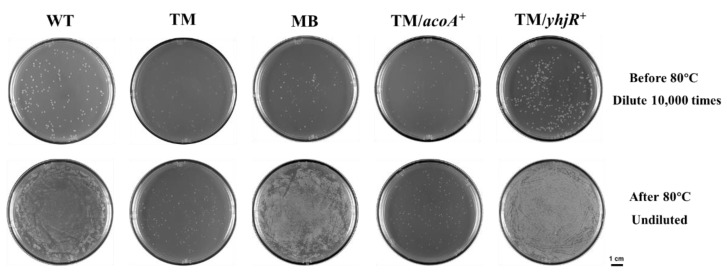
The sporulation ability in WT, TM, MB, TM/*acoA*^+^, and TM/*yhjR*^+^. The strains of WT, TM, MB, TM/*acoA*^+^, and TM/*yhjR*^+^ were subjected to the high-temperature treatment at 80 °C and observed for spores. The control samples were diluted (10,000 times) before high-temperature treatment for plating. The treatment samples were undiluted after high-temperature treatment, plated on LB agar plates and incubated for 12 h at 37 °C. The images shown are the representatives of three independent experiments. The scale represents 1 cm.

Given sporulation is highly related to biofilm formation in *B. subtilis* [53], sporulation assays with the strains of WT, TM, MB, TM/*acoA*^+^ and TM/*yhjR*^+^ were performed. As shown in Figure 8, TM produced dramatically fewer spores than WT, after high-temperature treatments at 80 °C. In contrast, MB produced more spores than WT after treatments at 80 °C (Figure 8). These phenotypes in sporulation were reminiscent of those in biofilm formation found in TM and MB, compared with WT. Moreover, we found that the overexpression of acoA could not restore the sporulation defect in TM, whereas the overexpression of yhjR could increase the sporulation in TM even to the MB level (Figure 8), which confirmed the physiological function of YhjR in sporulation. Our data indicated that YhjR might be a key factor connecting tmRNA and sporulation and might be used as a checkpoint for functional sporulation affecting the downstream bioprocesses such as biofilm formation in *B. subtilis*. The transcriptome data also supported the tight relationship between tmRNA and sporulation, given that most of the known sporulation-related genes were differentially expressed in TM compared with WT, and most of these DEGs showed restored expression in MB (Table S10). Obviously, studies in the molecular mechanism by which tmRNA mediates sporulation in *B. subtilis* will shed light on how tmRNA affects the physiology in this organism.

## 4. Discussion

In this study, we investigated the effect of tmRNA on biofilm formation in *B. subtilis*, which has not been described in *Bacillus* before. First, we constructed the *ssrA*-clean-deletion mutant (TM), using the CRISPR-Cas9 system in *B. subtilis* NCIB 3610. The results showed that TM produced remarkably less biofilms than WT (Figure 1), and showed a severe defect in swarming compared with WT (Figure 2a). We then isolated swarming suppressors of *ssrA* and found that one of these suppressors, tm3MB (MB), possessed not only a restored but even a reinforced biofilm-formation phenotype compared with WT (Figure S3 and Figure 2b,c). Thus, we considered the MB strain as a biofilm-formation suppressor of *ssrA* and performed various assays with these strains, to elucidate the possible mechanism(s) underlying the tmRNA regulation of biofilm formation in *B. subtilis*.

We analyzed the gene-expression differences among WT, TM, and MB by comparing the transcriptome-sequencing results of these strains. Compared with WT, a large number of genes were differentially expressed in TM, and the DEGs covers many cellular processes including biofilm-related ones (Figure 3). Compared with TM, the spontaneous suppressor mutation(s) in MB caused differential expression of many genes in this strain (Figure 4). Most of the DEGs found in the TM vs. WT comparison had restored expression to the WT level in the MB strain, indicating that the suppressor mutation(s) in MB were highly related to tmRNA in *B. subtilis*. Compared with WT, MB possessed fewer DEGs than TM (Figure 5a), and most of the DEGs were the ones resulting from the tmRNA deletion (Figure 5b). However, there were DEGs that appeared to be MB-specific, such as the genes grouped into the ribosome metabolism and amyloid fiber biosynthesis (Figure 5b,d). Unfortunately, simply grouping the DEGs based on KEGG annotation and biofilm-related bioprocesses did not provide sufficiently precise information to target pathway(s) mediating the tmRNA regulation of biofilm formation. Thus, we focused on particular DEGs that were most regulated in the comparisons. Finally, two sets of DEGs with special expression patterns were found in WT, TM, and MB. The first set includes *acoA*, *acoL*, *gerPA*, *ydgA*, *yhjR*, *yngJ*, *ytcB*, and *yxbB*, whose expression is regulated in a similar manner by which biofilm formation is regulated based on the comparison of WT, TM, and MB (Tables S4, S5 and S7). The second set includes *hxlB* and *rocA*, whose expression is regulated in the opposite manner by which biofilm formation is regulated based on the comparison of WT, TM, and MB (Tables S3 and S6). These genes could be considered as potential intermediate activators and repressors of biofilm formation in the tmRNA regulatory cascade(s) in *B. subtilis*.

Furthermore, the roles of known biofilm-related factors EPS and SinR were tested. The results showed that addition of EPS could restore the biofilm formation in TM to a WT level (Figure 6), suggesting that tmRNA regulated biofilm formation by affecting EPS production. However, the genes involved in EPS or other extracellular matrix structures were regulated in an unclear manner that could not explain the phenotypes found for the biofilm formation and EPS production in TM, compared with WT (Table S9). This might be because the EPS-related genes were differentially expressed at post-transcriptional levels or that there were unknown tmRNA-controlled processes dedicated to EPS production in *B. subtilis*. In addition, our data showed that even the expression of *sinR* was slightly up-regulated in TM and down-regulated in MB, compared with WT. Knocking out the *sinR* gene in TM did not restore its biofilm-formation defect (Figure S6), which indicates that the tmRNA regulation of biofilm formation in *B. subtilis* is SinR-independent. Thus, studies seeking for novel pathways involved in the tmRNA regulatory network mediating biofilm formation are essential.

To elucidate the unknown factors connecting tmRNA and biofilm formation in *B. subtilis*, we tested the effects of several genes (*acoA*, *acoL*, *gerPA*, *ydgA*, *yhjR*, *yngJ*, *ytcB*, and *yxbB*) whose special expression pattern has been described above. The data show that overexpressing *acoA* could partially restore the biofilm defect in TM (Figure 7), and the *acoA* mutants showed delayed biofilm formation compared with the parent strains (Figure S8), confirming the role of AcoA in the tmRNA regulation of biofilm formation. Moreover, the overexpression of *yhjR* largely restored the biofilm-formation defect in TM (Figure 7), indicating its role in connecting tmRNA and biofilm formation in *B. subtilis*. The *acoABCL* operon encodes the acetoin dehydrogenase complex, and the *yhjR* gene encodes spore coat protein. Thus, tmRNA might control biofilm formation through different pathways including acetoin metabolism and sporulation. In fact, we observed that sporulation was obviously affected in TM and MB, compared with WT, in a similar manner to that observed for biofilm formation (Figure 8). The overexpression of *yhjR* caused greatly restored sporulation in TM (Figure 8), which further confirmed the role of YhjR in the tmRNA regulatory network. It is possible that YhjR was used as a checkpoint for successful sporulation which positively affected biofilm formation in *B. subtilis*. However, unveiling the full web comprising tmRNA, acetoin metabolism, sporulation, and biofilm formation definitely requires more investigation in our future studies.

Despite the initial findings for the molecular mechanism by which tmRNA regulates biofilm formation in *B. subtilis*, we are far from a clear understanding of the whole tmRNA regulatory network in this bacterium. To accomplish this, we can use the following strategies in the future studies: (1) Given that tmRNA catalyzes trans-translation that is dedicated to rescuing stalled ribosomes, it might affect the proteome more directly than the transcriptome in bacteria. Thus, we can investigate the proteome patterns in WT, TM, MB, and other TM derivates, which might provide us more precise information about the tmRNA regulation. (2) We have identified the suppressor mutations in MB, which were listed in Figure S9 and Table S11, and further analyses of these mutations are required because the existence of them restored the biofilm-formation defect resulting from the tmRNA deletion. In addition, isolation and characterization of more biofilm suppressors of *ssrA* could be useful. (3) We should continue investigating the transcriptome results obtained in this study, especially the DEGs with an expression pattern similar or opposite to that for *acoA* and *yhjR* in WT, TM, and MB (Figure S10), because these findings about the roles of AcoA and YhjR in the tmRNA effect on biofilm formation provided us insights into tmRNA and its regulation in *B. subtilis*. (4) It has been reported that some compounds, such as surfactin, bacillomycin D, and bacillaene, can promote biofilm formation in *B. subtilis* [54,55,56], and we found that there were some compound-related genes, such as the ones encoding sporulation killing factor, bacillaene, bacilysin, plipastatin, and CDPS, down-regulated in TM, compared with WT, and the expression of most of these genes was restored in MB to a WT level (Table S12 and Figure S11). These results indicate that the reduction of biofilm formation in TM might be related to compound production, but that the enhancement of biofilm formation in MB might not be related to compound production. Furthermore, we found that the deletion of *skfA* that encodes a sporulation killing factor caused impaired biofilm formation in WT and MB (Figure S12), which partially supported our hypothesis about the relationship between compound production and biofilm formation, which were both regulated by tmRNA. Thus, investigation of the functions of particular compounds in the tmRNA regulation of biofilm formation will also be a promising research direction.

## 5. Conclusions

In conclusion, we demonstrated that the tmRNA deletion caused a biofilm-formation defect in *B. subtills* NCIB 3610. We successfully isolated a biofilm suppressor of *ssrA*, designated as MB, that could form thicker and more complex biofilms even than the WT strain. By analyzing the transcriptome data for WT, TM, and MB, we finally located two genes, *acoA* and *yhjR*, that could positively affect biofilm formation in *B. subtilis*, given that the overexpression of these genes caused restored phenotypes in biofilm formation in TM. We also conducted an initial characterization of the close relationship among tmRNA, sporulation, and biofilm formation in *B. subtilis*. Our data contribute more knowledge to the molecular regulatory network of biofilm formation in *B. subtilis* and may provide novel targets for better utilizing and modifying biofilm formation in *Bacillus* strains.

## Data Availability

The RNA sequence data have been deposited in the GEO database of NCBI with the accession code GSE199151. The whole-genome sequencing data that support the findings of this study have been deposited in the Sequence Read Archive with the project number PRJNA817865.

## Supplementary Materials

The following supporting information can be downloaded at: https://www.mdpi.com/article/10.3390/microorganisms10071338/s1, Figure S1: Schematic illustration of the deletion for ssrA (left) and PCR validation (right); Figure S2: Comparison of the biofilm formation among WT, TM, and CΔssrA; Figure S3: Isolation of suppressor(s) of ssrA and comparison of the biofilm formation among WT, TM, and isolated strains; Figure S4: The quality of the RNA samples and qPCR validation of transcriptome data. Figure S5: A Venn diagram showing the distribution of DEGs in comparisons WT vs. TM, TM vs. MB, and WT vs. MB; Figure S6: Comparison of the biofilm formation among WT, TM, and TM/ΔsinR; Figure S7: Comparison of the biofilm formation among WT/33N, TM/33N, and overexpression strains; Figure S8: Comparison of the biofilm formation among WT, WT/ΔacoA, MB, and MB/ΔacoA; Figure S9: Diagrams showing where the suppressor mutations were located and the consequences of these mutations in MB; Figure S10: Trend analysis of DEGs; Figure S11: Transcriptome analysis of genes related to compound biosynthesis pathways; Figure S12: Comparison of the biofilm formation among WT, WT/ΔskfA, MB, and MB/ΔsfkA.; Table S1: Strains and plasmids used in this study; Table S2: Primers used in this work; Table S3: The most up-regulated genes comparing TM with WT; Table S4: The most down-regulated genes comparing TM with WT; Table S5: The most up-regulated genes comparing MB with TM; Table S6: The most down-regulated genes comparing MB with TM; Table S7: The most up-regulated genes comparing MB with WT; Table S8: The most down-regulated genes comparing MB with WT; Table S9: The biofilm-related genes differentially expressed among strains WT, TM, and MB; Table S10: The sporulation-related genes differentially expressed among strains WT, TM, and MB; Table S11: The expression of genes spontaneously mutated in the MB strain; Table S12: The expression of genes related to secondary metabiotic products in strains WT, TM, and MB. Reference [57] is cited in the Supplementary Materials.
