# Supplementary material for: Transcriptomic Analysis Reveals the Role of tmRNA on Biofilm Formation in Bacillus subtilis
Source: Microorganisms. 2022 Jul 1;10(7):1338. doi: 10.3390/microorganisms10071338 (PMC9319509; doi:10.3390/microorganisms10071338)
Supplement: Supplementary file 1 [file microorganisms-10-01338-s001.zip › microorganisms-1787444-supplementary.pdf]

Transcriptomic analysis reveals the role of tmRNA on biofilm formation in *Bacillus subtilis*

Shanshan Xu [1,2], Qianqian Cao [1], Zengzhi Liu[1], Junpeng Chen [1], Peiguang Yan [2], Bingyu Li [3,*] Ying Xu [1,*]

[1] Shenzhen Key Laboratory of Marine Bioresource and Eco-Environmental Science, Shenzhen Engineering Laboratory for Marine Algal Biotechnology, College of Life Sciences and Oceanography, Shenzhen University, Shenzhen 518055, China

[2] College of Physics and Optoelectronic Engineering, Shenzhen University, Shenzhen 518060, China

[3] Guangdong Key Laboratory for Genome Stability and Disease Prevention, Health Science Center, Shenzhen University, Shenzhen, China

*Correspondence: Ying Xu, boxuying@szu.edu.cn; Bingyu Li, byli1988@foxmail.com

Supplementary Materials: Supplementary Figures S1-S11, Supplementary Tables S1-S12.

Supplementary Figures

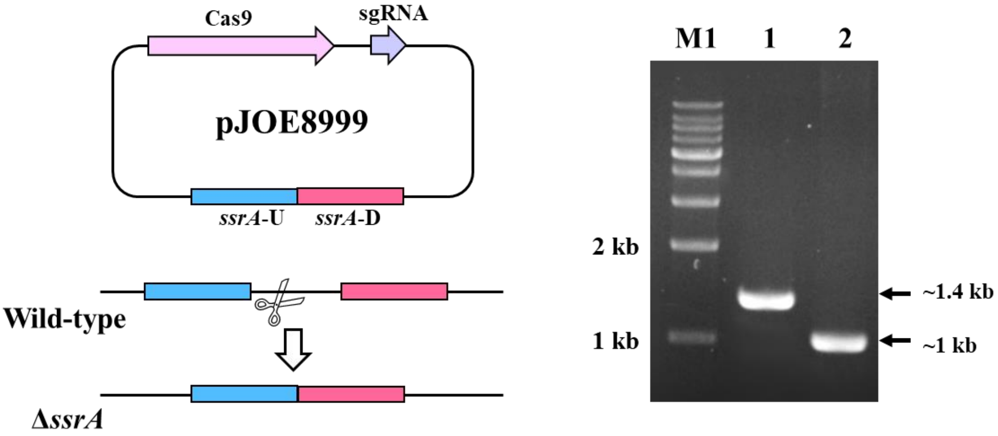

Supplementary Figure S1. Schematic illustration of the deletion for ssrA (left) and PCR validation (right). Lane M1, 1 kb DNA ladder marker; Lane 1, PCR product amplified from the wild-type strain; Lane 2, PCR product amplified from the Δ*ssrA* strain.

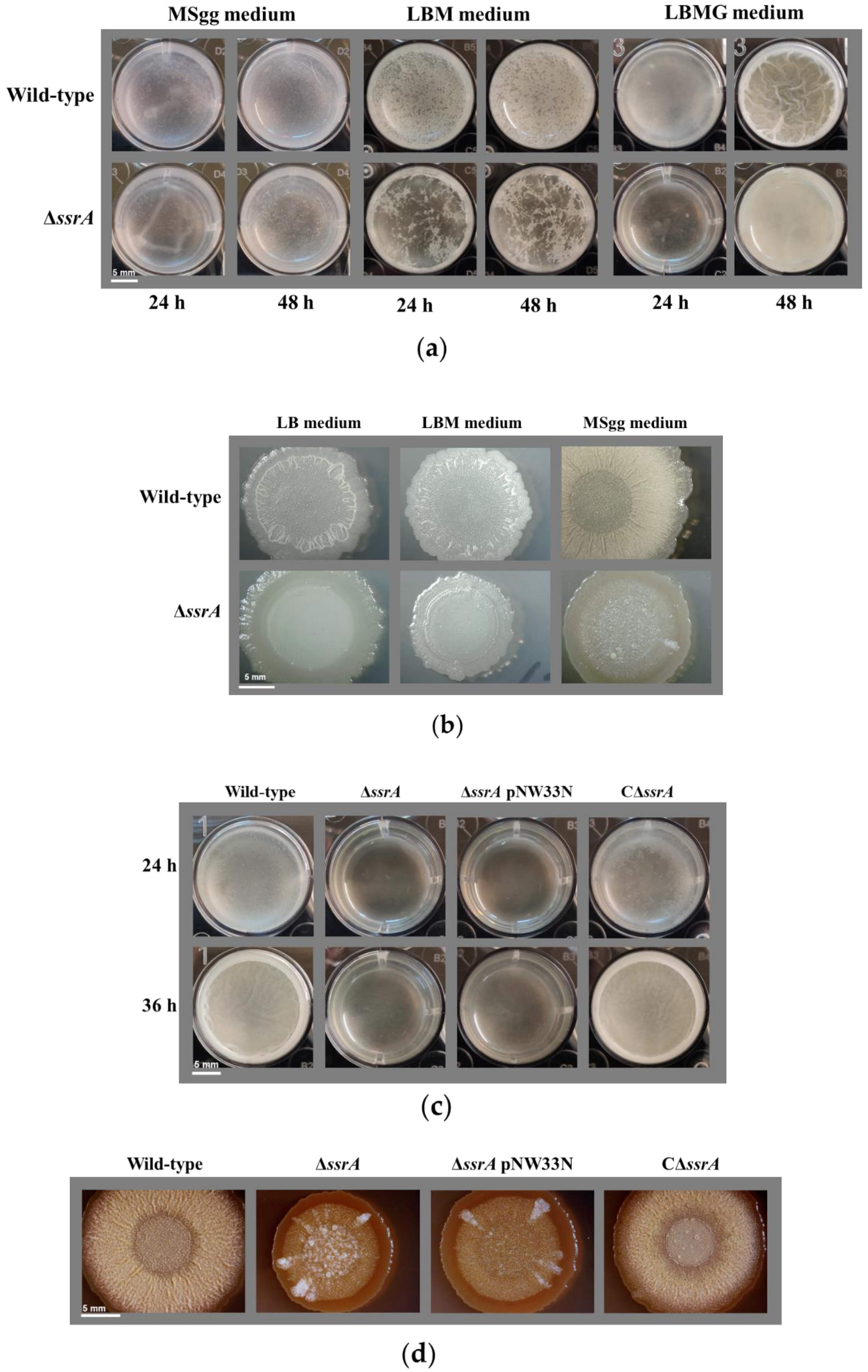

Supplementary Figure S2. Comparison of the biofilm formation among WT, TM, and CΔ*ssrA*.

(a) The wild-type strain (WT) and *ssrA* mutant (TM) were grown in MSgg, LBM and LBMG liquid medium at 30°C and observed for biofilm formation at different times as indicated. The scale bar represents 5 mm. The shown images are the representatives of three independent experiments. (**b)** WT and TM were grown in LB, LBM and MSgg agar plates at 30°C and observed for biofilm formation after 3-day incubation. The scale bar represents 5 mm. The shown images are the representatives of three independent experiments. (**c)** WT, TM, TM/pNW33N, and CΔ*ssrA* (TM/pNW33N-*ssrA*) were grown in the LBMG liquid medium at 30°C and observed for biofilm formation at different times as indicated. The scale bar represents 5 mm. The shown images are the representatives of three independent experiments. (**d)** WT, TM, TM/pNW33N, and CΔ*ssrA* (TM/pNW33N-*ssrA*) were grown in the MSgg agar plates at 30°C for 3 days and observed for biofilm formation. The scale bar represents 5 mm. The shown images are the representatives of three independent experiments.

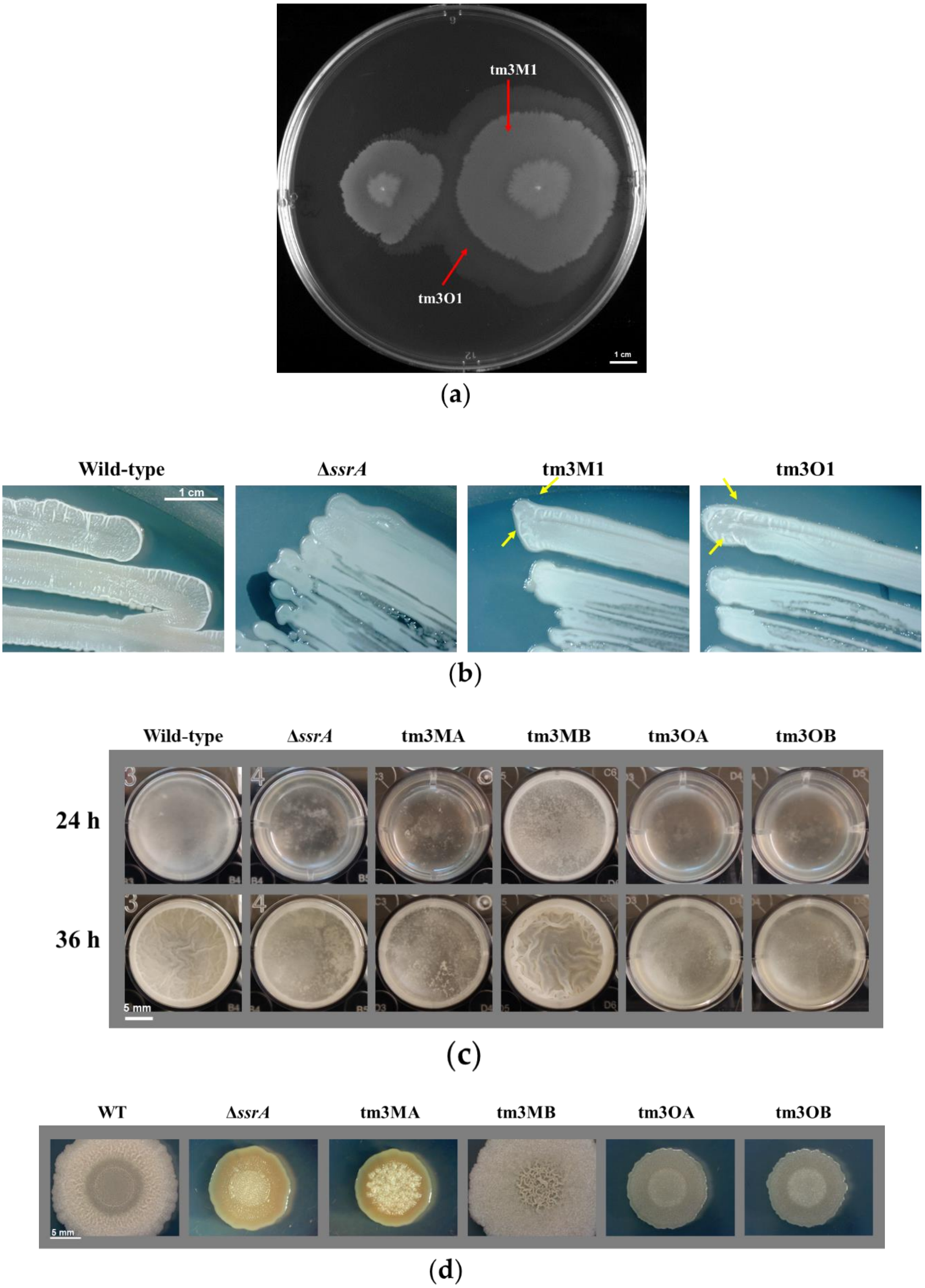

Supplementary Figure S3. Isolation of suppressor(s) of *ssrA* and comparison of the biofilm formation among WT, TM, and isolated strains. (a) To isolate putative swarming suppressors of *ssrA*, TM was grown in soft agar plates until obvious swarming zones appeared. The scale bar represents 1 cm. The red arrows indicated the areas where bacteria were picked for further purification of suppressors. **(b)**

Bacteria of WT, TM, tm3M1, and tm3O1 were grown in LB agar plates (1.5% agar) for colony-morphology observation. The yellow arrows indicated the wrinkled colony edges of tm3M1 and tm3O1. The scale bar represents 1 cm. (**c)** WT, TM, tm3MA, tm3MB, tm3OA, and tm3OB were grown in the LBMG liquid medium at 30°C and observed for biofilm formation at different times as indicated. The scale bar represents 5 mm. The shown images are the representatives of three independent experiments. tm3MA and tm3MB are strains purified from bacteria of tm3M1. tm3OA and tm3OB are strains purified from bacteria of tm3O1. **(d)** WT, TM, tm3MA, tm3MB, tm3OA, and tm3OB were grown in the MSgg agar plates at 30°C for 3 days and observed for biofilm formation. The scale bar represents 5 mm. The shown images are the representatives of three independent experiments.

(a)

No.	Sample Name	Concentration(ng/ul)	RIN number
1	WT-1	1315	10.00
2	WT-2	2545	10.00
3	WT-3	2411	10.00
4	TM-1	921	9.60
5	TM-2	1483	9.90
6	TM-3	1598	9.80
7	MB-1	942	10.00
8	MB-2	1844	9.90
9	MB-3	1005	10.00

(b)

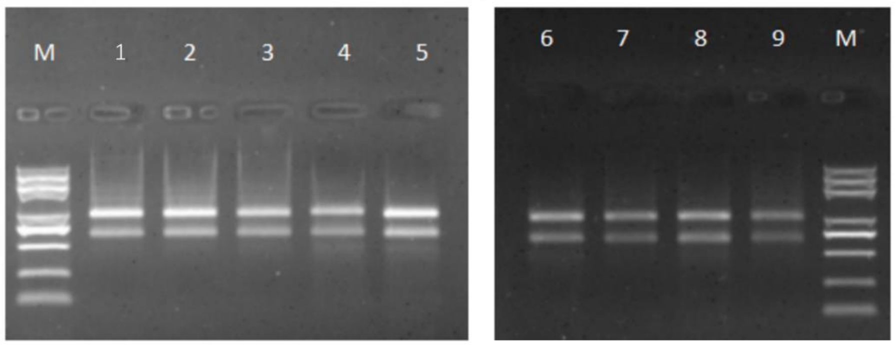

(c)

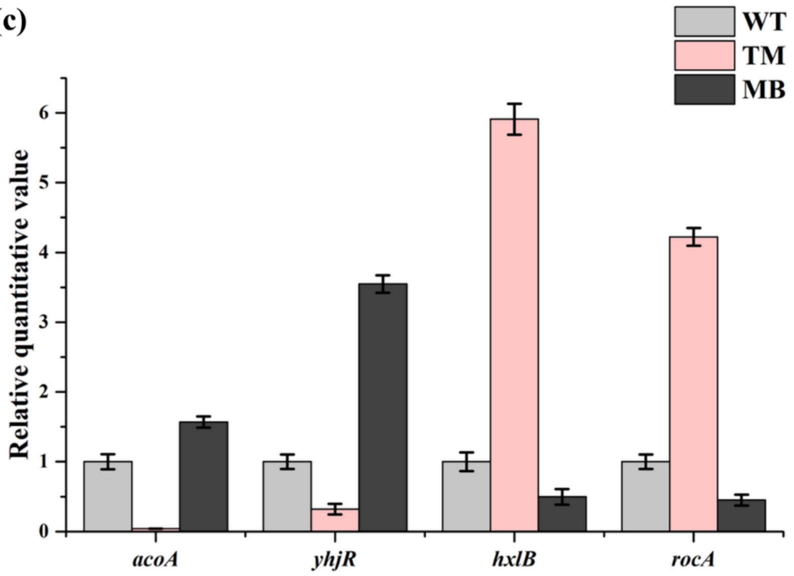

Supplementary Figure S4. The quality of the RNA samples and qPCR validation of transcriptome data. (a) The RIN number of the RNA samples. (b) Gel electropherogram of RNA samples. (c) qPCR validation of target genes. The result was consistent with transcriptome data.

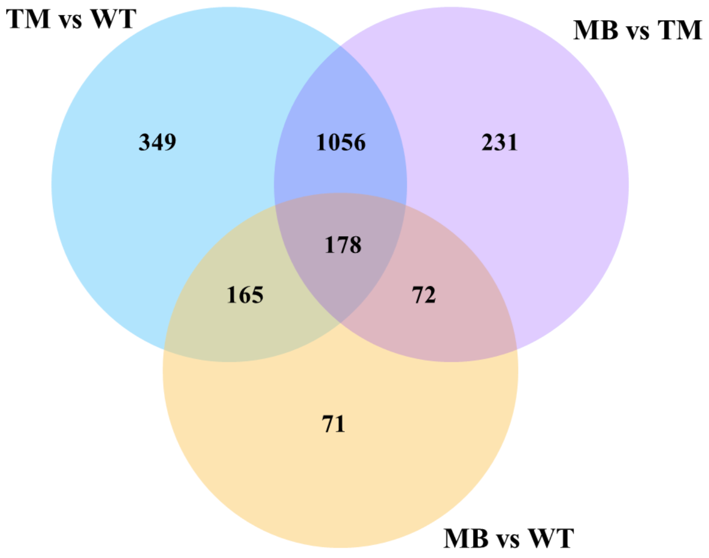

Supplementary Figure S5. A venn diagram showing the distribution of DEGs in comparisons of WT *vs.* TM, TM *vs.* MB, and WT *vs.* MB.

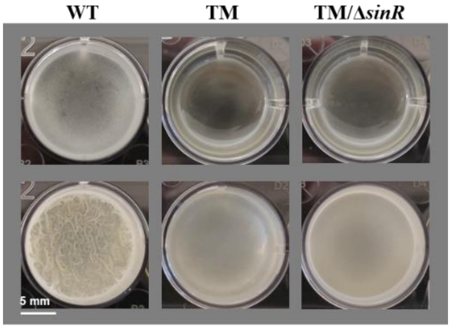

(a)

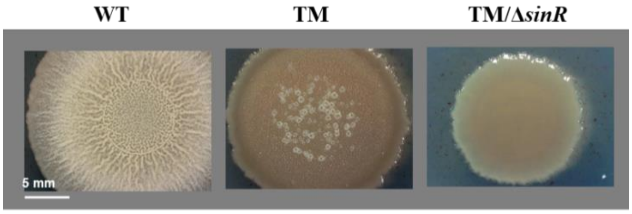

(b)

Supplementary Figure S6. Comparison of the biofilm formation among WT, TM, and TM/Δ*sinR*. **(a)** WT, TM, and TM/Δ*sinR* were grown in the LBMG liquid medium at 30°C and observed for biofilm formation at different times as indicated. The scale bar represents 5 mm. The shown images are the representatives of three independent experiments. **b** WT, TM, and TM/Δ*sinR* were grown in the MSgg agar plates at 30°C for 3 days and observed for biofilm formation. The scale bar represents 5 mm. The shown images are the representatives of three independent experiments.

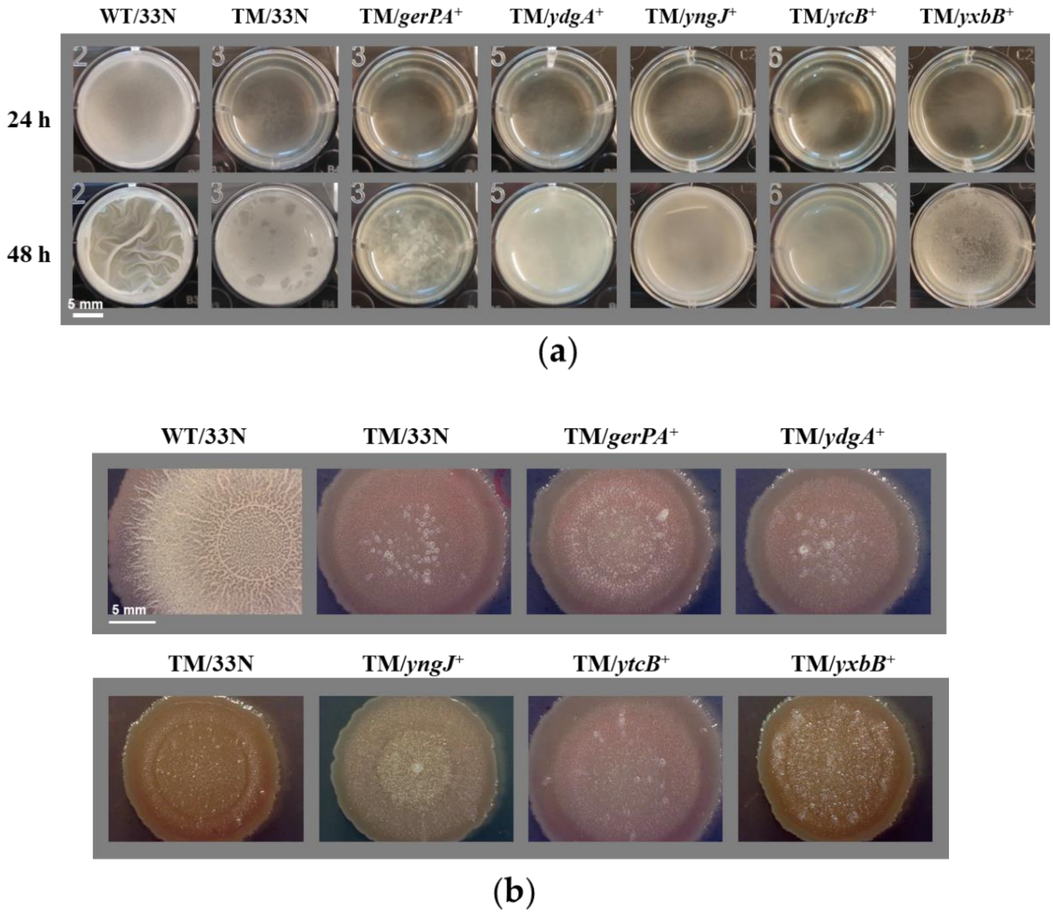

Supplementary Figure S7. Comparison of the biofilm formation among WT/33N, TM/33N, and overexpression strains. (a) WT/33N, TM/33N and overexpression strains were grown in the LBMG liquid medium at 30°C and observed for biofilm formation at different times as indicated. The scale bar represents 5 mm. The shown images are the representatives of three independent experiments. (**b)** WT/33N, TM/33N and overexpression strains were grown in the MSgg agar plates at 30°C for 3 days and observed for biofilm formation. The scale bar represents 5 mm. The shown images are the representatives of three independent experiments.

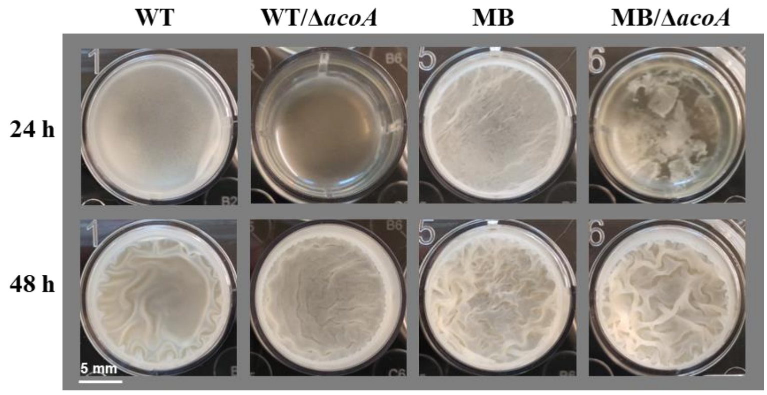

(**a**)

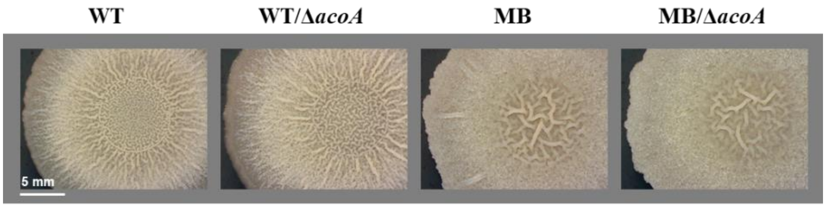

(**b**)

Supplementary Figure S8. Comparison of the biofilm formation among WT, WT/Δ*acoA*, MB, and MB/Δ*acoA*. (a) WT, WT/Δ*acoA*, MB, and MB/Δ*acoA* were grown in the LBMG liquid medium at 30°C and observed for biofilm formation at different times as indicated. The scale bar represents 5 mm. The shown images are the representatives of three independent experiments. (**b**) WT, WT/Δ*acoA*, MB, and MB/Δ*acoA* were grown in the MSgg agar plates at 30°C for 3 days and observed for biofilm formation. The scale bar represents 5 mm. The shown images are the representatives of three independent experiments.

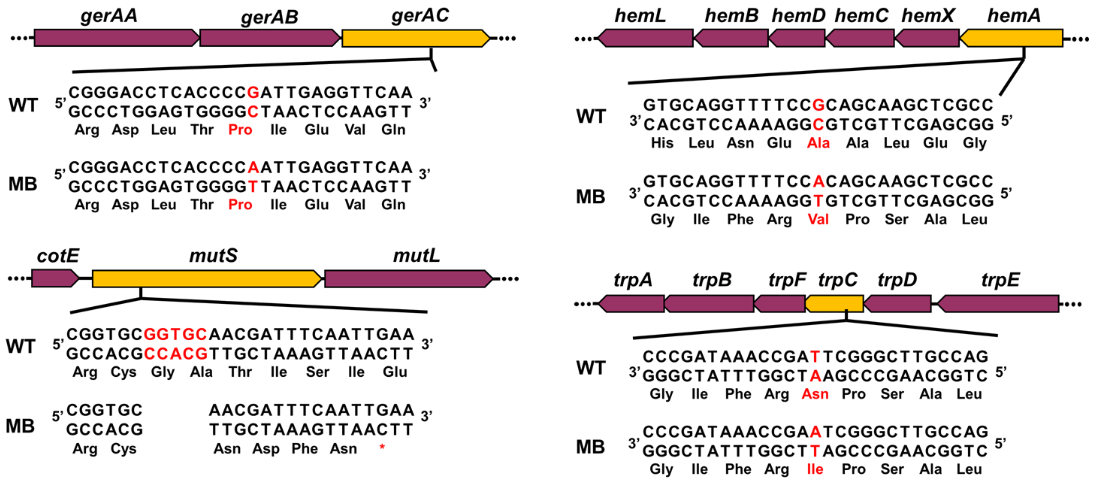

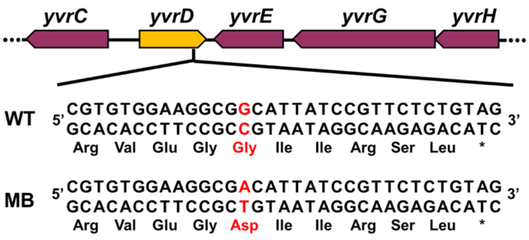

Gene	Base mutation	Amino acid mutation
gerAC	CCG → CCA	Pro_{217} → Pro_{217}
hemA	GCG → GAG	Ala_{198} → Glu_{198}
mutS	lack of GGTGC	frame shift mutation
trpC	AAT → ATT	Asn_{37} → Ile_{37}
yvrD	GGC → GAC	Gly_{258} → Asp_{258}

Supplementary Figure S9. Diagrams showing where the suppressor mutations were located and the consequences caused by these mutations in MB.

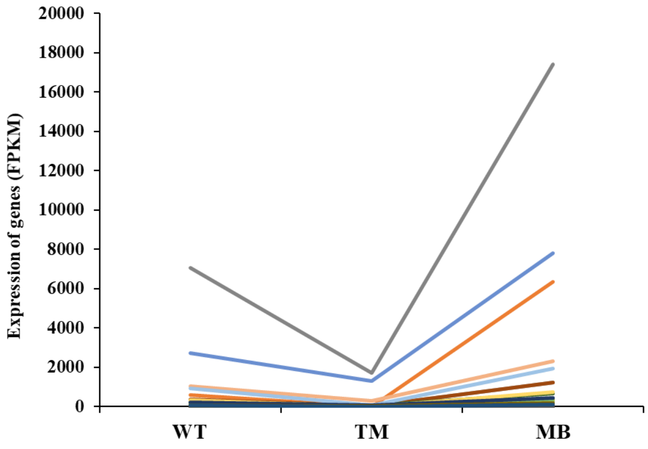

(**a**)

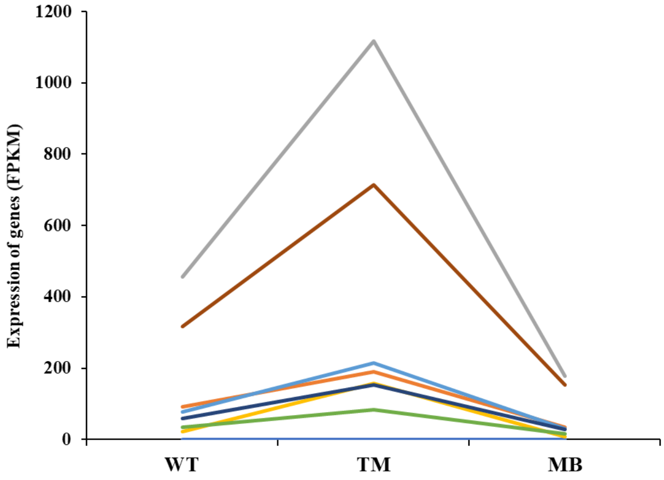

(**b**)

Supplementary Figure S10. Trend analysis of DEGs. (a) A chart showing the expression of DEGs with an *acoA*-like pattern in WT, TM, and MB. (**b)** A chart showing the expression of DEGs with an *acoA*-opposite pattern in WT, TM, and MB.

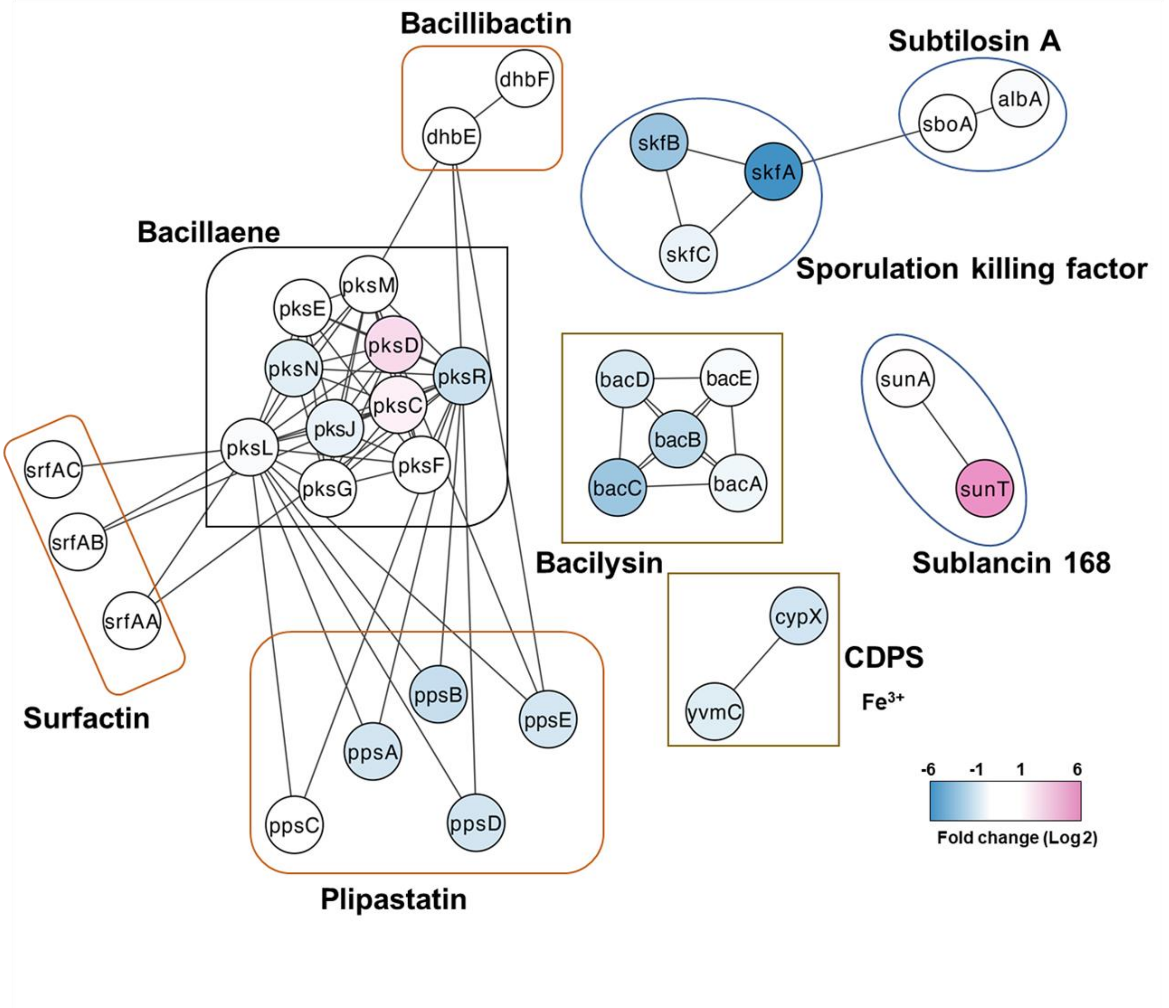

Supplementary Figure S11. Transcriptome analysis of genes related to compound biosynthesis pathways. Blue circles represent the down-regulated genes comparing TM with WT, and pink circles represent the up-regulated genes comparing TM with WT. Gene function and biosynthetic gene cluster attribution was confirmed using SubtiWiki database [49]. The protein-protein interaction network diagram was generated by the STRING service (https://string-db.org/). The network diagrams were annotated and colored by Cytoscape v3.9.1 [1].

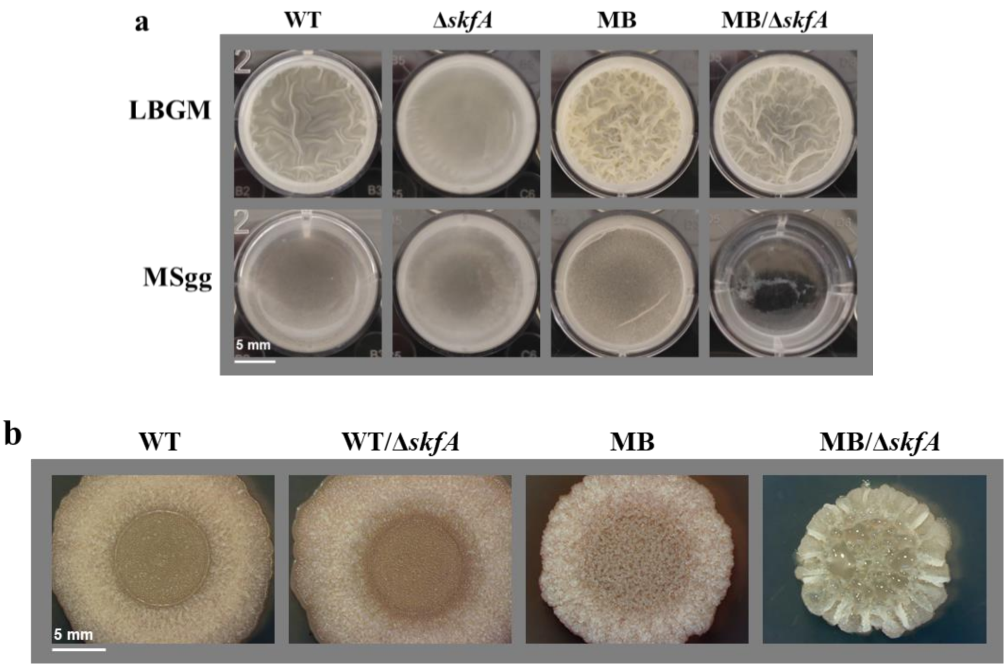

Supplementary Figure S12: Comparison of the biofilm formation among WT, WT/Δ*skfA*, MB, and MB/Δ*sfkA*. a WT, WT/Δ*skfA*, MB, and MB/Δ*skfA* were grown in the LBMG and MSgg liquid media at 30°C and observed for biofilm formation at different times as indicated. The scale bar represents 5 mm. The shown images are the representatives of three independent experiments. **b** WT, WT/Δ*skfA*, MB, and MB/Δ*skfA* were grown in the MSgg agar plates at 30°C for 3 days and observed for biofilm formation. The scale bar represents 5 mm. The shown images are the representatives of three independent experiments.

Supplementary Tables

Supplementary Table S1: Strains and plasmids used in This study.

**Strain name**	**Parent**	**Genotype or Characteristic**	**Source**
DH5α		An *E. coli* strain for molecular cloning	Lab stock
JM110		An *E. coli* strain for modified plasmids for Bacillus transformation, Str^R	Lab stock
WT	3610	Wild-type strain	Lab stock
TM	3610	The *ssrA*-double-crossingover mutant in the 3610 wild-type strain	This study
MB	TM	The biofilm-formation suppressor of *ssrA*	This study
CΔ*ssrA*	TM	pNW33N::P_{ssrA}-*ssrA* (pYX202), Δ*ssrA* Chl^R; the *ssrA* complementary strain	This study
WT/pNW33N	3610	pNW33N (pYX203), Chl^R; as a control for biofilm formation assay	This study
TM/pNW33N	TM	pNW33N (pYX203), Chl^R; as a control for biofilm formation assay	This study
TM/*acoA*$^+$	TM	pNW33N::pG-*acoA* (pYX241), Δ*ssrA* Chl^R; *acoA* gene overexpression in TM	This study
TM/*yhjR*$^+$	TM	pNW33N:: pG-*yhjR* (pYX251), Δ*ssrA* Chl^R; *yhjR* gene overexpression in TM	This study
Δ*acoA*	3610	The *acoA*-double-crossingover mutant in WT	This study
Δ*yhjR*	3610	The *yhjR*-double-crossingover mutant in WT	This study
MBΔ*acoA*	MB	The *acoA*-double-crossingover mutant in MB	This study
MBΔ*yhjR*	MB	The *yhjR*-double-crossingover mutant in MB	This study
Δ*skfA*	3610	The *skfA*-double-crossingover mutant in WT	This study
MBΔ*skfA*	MB	The *skfA*-double-crossingover mutant in MB	This study
Δ*sinR*	3610	The *sinR*-double-crossingover mutant in WT	This study
TMΔ*sinR*	TM	The *sinR*-double-crossingover mutant in MB	This study
**Plasmid name**	**Background**	**Genotype or Characteristics**	**Source**

pJOE8999	DH5α	A temperature-sensitive shuttle vector for *B. subtilis* to knock out genes with Crispr-Cas9, Kan^R	[34]
pYX101	DH5α	pJOE8999::*ssrA*-gRNA, Kan^R	This study
pYX102	DH5α	pJOE8999::*ssrA*-gRNA::*ssrA*-UD, Kan^R	This study
pYX103	JM110	pJOE8999::*ssrA*-gRNA::*ssrA*-UD, Kan^R	This study
pNW33N	DH5α	*E.coli* – Gram+ shuttle vector, Chl^R	*Bacillus* Genetic Stock Center
pYX201	DH5α	pNW33N::P*ssrA*-*ssrA*, Chl^R	This study
pYX202	JM110	pNW33N::P*ssrA*-*ssrA*, Chl^R	This study
pYX203	JM110	pNW33N, Chl^R	This study
pYX240	DH5α	pNW33N::pG-*acoA*, Chl^R	This study
pYX241	JM110	pNW33N::pG-*acoA*, Chl^R	This study
pYX250	DH5α	pNW33N::pG-*yhjR*, Chl^R	This study
pYX251	JM110	pNW33N::pG-*yhjR*, Chl^R	This study

Abbreviations for the antibiotic resistance genes: Kan, kanamycin; Str, Streptomycin; Chl, chloramphenicol.

Supplementary Table S2: Primers used in This work. (The underlined part represents the restriction-enzyme sites)

**Name**	**Sequence (5' – 3')**	**Built-in Restriction-Enzyme Sites**	**Usefulness**
ssrA-gRNA-F<br>*ssrA*-gRNA-R	TACGTGTGAAGAGCACATCCAAGT<br>AAACACTTGGATGTGCTCTTCACA	*Bsa* I	gRNA of *ssrA*, for double crossing over mutant construction
ssrA-U-F<br>*ssrA*-U-R	AAGGCCAACGAGGCCAAAGACACTGCGGACCAAA<br>CCCGTATTTCCCTTAACTCCCACCGTCTCCATAC	*Sfi* I	Upstream fragment of *ssrA*, for double crossing over mutant construction
ssrA-D-F<br>*ssrA*-D-R	GGAGACGGTGGGAGTTAAGGGAAATACGGGAGAA<br>AAGGCCTTATTGGCCCAATAAAGATGAGCGCAATA	 *Sfi* I	Downstream fragment of *ssrA* *ssrA*, for double crossing over mutant construction
ssrA-verify-F<br>*ssrA*-verify-R	AAATCCCTAAATTGTTGACC<br>TTCTGCAAGGAACCGAAAT		Verifying the *ssrA* mutants
ssrA-33n-F<br>*ssrA*-33n-R	AACTGCAGAGGCATAGTGCTTGATTCG<br>CGGGATCCGTATGGAGACGGTGGGAGT	*Pst* I<br>*Bam*H I	Cloning *ssrA* in pNW33N for construction of the complementary strain
pG-gibson-F<br>pG-gibson-R	CCAAGCTTGCATGCCTGCAGGCTCCAGTATTCTGACATGG<br>TAATATCGCCTCCTATTGTAA		Cloning pG region in pNW33N
acoA-gibson-F<br>*acoA*-gibson-R	TACAATAGGAGGCGATATTAATGAAATTGTTAAAACGAGA<br>AGCTCGGTACCCGGGGATCCTATGACTCTCGCCATCTCTT		Cloning the *acoA* gene in pNW33N
yhjR-gibson-F<br>*yhjR*-gibson-R	TACAATAGGAGGCGATATTAATGGATAATTTTAAATTAGA<br>AGCTCGGTACCCGGGGATCCAGACAAGGCTTTTCGCCTTG		Cloning the *yhjR* gene in pNW33N
33n-verify-FP<br>33n-verify-RP	GAGCGAGGAAGCGGAAGAG<br>TTTGAAGGGCTGGCACGAC		Verifying the over-expression plasmids
gerPA-gibson-F<br>gerPA-gibson-R	TACAATAGGAGGCGATATTAATGCCGGCCATTGTCGGAG<br>AGCTCGGTACCCGGGGATCCCTTTCATATGATTAACGGCC		Cloning *gerPA* gene to pNW33N
ydgA-gibson-F<br>ydgA-gibson-R	TACAATAGGAGGCGATATTAATGCCATATCAAATTAATAT<br>AGCTCGGTACCCGGGGATCCTTCTCCTTAAATTTGATTTG		Cloning *ydgA* gene to pNW33N
yngJ-gibson-FP	TACAATAGGAGGCGATATTAATGAATTTTGAGCTGACAAA		Cloning *yngJ* gene to pNW33N

yngJ-gibson-RP	AGCTCGGTACCCGGGGATCCCCTCCTTTCTTAATGATAGG		
ytcB-gibson-F<br>ytcB-gibson-R	TACAATAGGAGGCGATATTAATGAAAATACTCGTCACAGG<br>AGCTCGGTACCCGGGGATCCTTATTCCCCCTGATACAGCG		Cloning *ytcB* gene to pNW33N
yxbB-gibson-F<br>yxbB-gibson-R	TACAATAGGAGGCGATATTAATGATTACGGCTGAAGAATG<br>AGCTCGGTACCCGGGGATCCATTGATCATTACAATGCACC		Cloning *yxbB* gene to pNW33N
acoA-gRNA-F<br>*acoA*-gRNA-R	TACGAACCAAGGTACCTTCCACGA<br>AAACTCGTGGAAGGTACCTTGGTT	*Bsa* I	gRNA of *acoA*, for double crossing over mutant construction
acoA-U-F<br>*acoA*-U-R	AAGGCCTTATTGGCCCCGCTATCATCATACAAAG<br>GCTGACACGCTGTTCAGCCCGTAAAATCAGTCTTC	*Sfi* I	Upstream fragment of *acoA*, for double crossing over mutant construction
acoA-D-F<br>*acoA*-D-R	ACTGATTTTACGGGCTGAACAGCGTGTCAGCGAATC<br>AAGGCCTTATTGGCCCAGCGACAAGCGCAGCAAT	*Sfi* I	Downstream fragment of *acoA*, for double crossing over mutant construction
acoA-verify-F<br>*acoA*-verify-R	TTAGACGCCAGCGGGTTAT<br>ATGTGGTGCGGACGGTAAT		Verifying the *acoA* mutants
yhjR-gRNA-F<br>*yhjR*-gRNA-R	TACGACACATCATGCCGAAACAAA<br>AAACTTTGTTTCGGCATGATGTGT	*Bsa* I	gRNA of *yhjR*, for double crossing over mutant construction
yhjR-U-F<br>*yhjR*-U-R	AAGGCCTTATTGGCCGGCATGTTCAGTATTTGTCG<br>ACTATCCCTATCCGGCACGCTGTCTGGTTCCTAT	*Sfi* I	Upstream fragment of *yhjR*, for double crossing over mutant construction
yhjR-D-F<br>*yhjR*-D-R	GAACCAGACAGCGTGCCGGATAGGGATAGTAGCT<br>AAGGCCAACGAGGCCGCAGGCGGTAATAGGTTTT	*Sfi* I	Downstream fragment of *yhjR*, for double crossing over mutant construction
yhjR-verify-F<br>*yhjR*-verify-R	GCTTGATGAAGAGGGATGG<br>TCCGCAGGAGCATTTGTAC		Verifying the *yhjR* mutants
skfA-gRNA-F<br>*skfA*-gRNA-R	TACGACTTCGATTGTGAAAGCTGC<br>AAACGCAGCTTTCACAATCGAAGT	*Bsa* I	gRNA of *skfA*, for double crossing over mutant construction
skfA-U-F<br>*skfA*-U-R	AAGGCCTTATTGGCCACCTGGGTTGGTGCGTTAG<br>CCCTATTCTCAAATGGCTCCCATGCGATACGATA	*Sfi* I	Upstream fragment of *skfA*, for double crossing over mutant construction
skfA-D-F<br>*skfA*-D-R	GTATCGCATGGGAGCCATTTGAGAATAGGGAGTT<br>AAGGCCAACGAGGCCCAATTATGTTCAGGCGATC	*Sfi* I	Downstream fragment of *skfA*, for double crossing over mutant construction

skfA-verify-F<br>*skfA*-verify-R	TTGCTCTGTTTCCGTTGTC<br>CGTAAATGGATGGGCACTA		Verifying the *skfA* mutants
sinR-gRNA-F<br>*sinR*-gRNA-R	TACGCGAAAAAGTCTCCGCTGTTC<br>AAACGAACAGCGGAGACTTTTTCG	*Bsa* I	gRNA of *sinR*, for double crossing over mutant construction
sinR-U-F<br>*sinR*-U-R	AAGGCCAACGAGGCCGAACCGCCCTTTCTACTGG<br>AGTGCCTCTGCTCAGTGTCATCACCTTCCTTGTG	*Sfi* I	Upstream fragment of *sinR*, for double crossing over mutant construction
sinR-D-F<br>*sinR*-D-R	AGGAAGGTGATGACACTGAGCAGAGGCACTAACT<br>AAGGCCTTATTGGCCATACAAAGGACAGCACCAT	*Sfi* I	Downstream fragment of *sinR*, for double crossing over mutant construction
sinR-verify-F<br>*sinR*-verify-R	TGCTTCACCATTTCACCTAT<br>GTAAAACCGCTCCTGAATAT		Verifying the *sinR* mutants

Supplementary Table S3: The most up-regulated genes comparing TM with WT

Gene ID	Gene name	Function	log2 (fold change)		
			**TM vs WT**	MB vs TM	MB vs WT
EJJ34_15785	*lrgA*	Antiholin-like protein LrgA	**6.90**	-2.65	4.21
EJJ34_15780	*lrgB*	Antiholin-like protein LrgB	**5.95**	-1.82	4.10
EJJ34_08550	*pyrB*	Aspartate carbamoyltransferase	**5.78**	-2.84	2.89
EJJ34_08560	*pyrAA*	Carbamoyl-phosphate synthase Pyrimidine-specific small chain	**5.03**	-3.61	1.38
EJJ34_08555	*pyrC*	Dihydroorotase	**4.93**	-3.02	1.86
EJJ34_22095	*yybN*	DUF2712 domain-containing protein	**4.84**	-2.48	2.32
EJJ34_05180	*cspB*	Cold-shock protein CspB	**4.82**	-3.31	1.46
EJJ34_08570	*pyrK*	Dihydroorotate oxidase B electron transfer subunit	**4.64**	-3.64	—
EJJ34_08565	*carB*	Carbamoyl-phosphate synthase pyrimidine-specific large chain	**4.62**	-3.72	—
EJJ34_08580	*pyrF*	Orotidine-5'-phosphate decarboxylase	**4.60**	-4.10	—
EJJ34_17300	*maeN*	Na^+-malate symporter	**4.60**	-4.51	—
EJJ34_08585	*pyrE*	Orotate phosphoribosyltransferase	**4.41**	-3.31	1.06
EJJ34_02035	*hxlA*	3-hexulose-6-phosphate synthase	**4.34**	-4.95	—
EJJ34_08575	*pyrD*	Dihydroorotate dehydrogenase	**4.11**	-3.50	—
EJJ34_11405	*yopT*	Hypothetical protein	**4.07**	-2.00	2.04
EJJ34_11780	*sunS*	SunS family peptide S-glycosyltransferase	**4.02**	-2.52	1.46
EJJ34_22110	*yybK*	DUF2705 domain-containing protein	**3.88**	-3.12	—
EJJ34_18065	*yvzF*	DUF3970 domain-containing protein	**3.81**	-3.26	—
EJJ34_11505	*yopB*	XRE family transcriptional regulator	**3.79**	—	3.75
EJJ34_22105	*yybL*	Hypothetical protein	**3.75**	-2.34	1.37

Supplementary Table S4: The most down-regulated genes comparing TM to WT

Gene ID	Gene name	Function	log2 (fold change)		
			**TM vs WT**	MB vs TM	MB vs WT
EJJ34_18365	*ssrA*	Transfer-messenger RNA	**-15.20**	—	-18.09
EJJ34_04560	*acoB*	Acetoin:2%2C6-dichlorophenolindophenol oxidoreductase subunit beta	**-8.89**	9.72	—
EJJ34_19650	*cotG*	Spore coat protein G	**-8.41**	5.09	-3.36
EJJ34_05115	*prkA*	Protein PrkA	**-8.20**	6.58	-1.66
EJJ34_12505	*spoIVA*	Stage IV sporulation protein A && -	**-7.86**	6.75	-1.14
EJJ34_15225	*safA*	Spore coat assembly protein SafA	**-7.78**	6.82	—
EJJ34_06265	*carA*	Carbamoyl-phosphate synthase arginine-specific small chain	**-7.71**	3.85	-3.90
EJJ34_15220	*coxA*	Sporulation cortex protein CoxA	**-7.61**	7.56	—
EJJ34_05910	*ntdB*	Kanosamine-6-phosphate phosphatase	**-7.59**	6.31	-1.33
EJJ34_05305	*yhdB*	Hypothetical protein	**-7.59**	8.07	—
EJJ34_05500	*sspB*	Small%2C acid-soluble spore protein B	**-7.59**	7.15	—
EJJ34_16790	***ytcB***	NAD-dependent epimerase/dehydratase family protein	**-7.54**	8.62	1.04
EJJ34_05905	*ntdC*	Glucose-6-phosphate 3-dehydrogenase	**-7.48**	6.38	-1.15
EJJ34_05790	*aprE*	Subtilisin E	**-7.46**	7.61	—
EJJ34_06540	*cotZ*	Spore coat protein Z	**-7.45**	7.86	—
EJJ34_06270	*carB*	Carbamoyl-phosphate synthase arginine-specific large chain	**-7.44**	3.93	-3.55
EJJ34_07915	*yknT*	Sporulation protein cse15	**-7.43**	7.14	—
EJJ34_06570	*yjzK*	Hypothetical protein	**-7.43**	7.23	—
EJJ34_04555	***acoA***	Acetoin:2%2C6-dichlorophenolindophenol oxidoreductase subunit alpha	**-7.38**	10.82	3.39
EJJ34_03975	*cotJA*	Protein CotJA	**-7.34**	7.60	—

Supplementary Table S5: The most up-regulated genes comparing MB with TM

Gene ID	Gene name	Function	log$_2$ (fold change)		
			TM vs WT	**MB vs TM**	MB vs WT
EJJ34_04555	***acoA***	Acetoin:2%2C6-dichlorophenolindophenol oxidoreductase subunit alpha	-7.38	**10.82**	3.39
EJJ34_04560	*acoB*	Acetoin:2%2C6-dichlorophenolindophenol oxidoreductase subunit beta	-8.89	**9.72**	—
EJJ34_16790	***ytcB***	NAD-dependent epimerase/dehydratase family protein	-7.54	**8.62**	1.04
EJJ34_05305	*yhdB*	Hypothetical protein	-7.59	**8.07**	—
EJJ34_04565	*acoC*	Dihydrolipoyllysine-residue acetyltransferase component of acetoin cleaving system	-7.31	**8.01**	—
EJJ34_04570	***lpdA/acoL***	Dihydrolipoyl dehydrogenase	-6.82	**7.95**	1.09
EJJ34_06540	*cotZ*	Spore coat protein Z	-7.45	**7.86**	—
EJJ34_05790	*aprE*	Subtilisin E	-7.46	**7.61**	—
EJJ34_03975	*cotJA*	Protein CotJA	-7.34	**7.60**	—
EJJ34_15220	*coxA*	Sporulation cortex protein CoxA	-7.61	**7.56**	—
EJJ34_06000	***gerPA***	Germination protein GerPA	-5.51	**7.32**	1.76
EJJ34_19910	*spoIIQ*	M23 family metallopeptidase	-7.25	**7.26**	—
EJJ34_06545	*cotY*	Spore coat protein Y	-6.80	**7.24**	—
EJJ34_06570	*yjzK*	Hypothetical protein	-7.43	**7.23**	—
EJJ34_05500	*sspB*	Small%2C acid-soluble spore protein B	-7.59	**7.15**	—
EJJ34_07915	*yknT*	Sporulation protein cse15	-7.43	**7.14**	—
EJJ34_09995	***yngJ***	Acyl-CoA dehydrogenase	-5.62	**7.12**	1.46
EJJ34_03220	***ydgA***	Spore germination protein	-5.68	**6.94**	1.22
EJJ34_10870	*yodT*	Aspartate aminotransferase family protein	-6.84	**6.86**	—
EJJ34_13355	*spoIIIAA*	Stage III sporulation protein AA	-7.13	**6.84**	—

Supplementary Table S6: The most down-regulated genes comparing MB with TM

Gene ID	Gene name	Function	log2 (fold change)		
			TM vs WT	**MB vs TM**	MB vs WT
EJJ34_02035	*hxlA*	3-hexulose-6-phosphate synthase	4.34	**-4.95**	—
EJJ34_17300	*maeN*	Na^{+}-malate symporter	4.60	**-4.51**	—
EJJ34_02030	***hxlB***	6-phospho-3-hexuloisomerase	2.85	**-4.19**	-1.37
EJJ34_08580	*pyrF*	Orotidine-5'-phosphate decarboxylase	4.60	**-4.10**	—
EJJ34_08565	*carB*	Carbamoyl-phosphate synthase pyrimidine-specific large chain	4.62	**-3.72**	—
EJJ34_20570	***rocA***	L-glutamate gamma-semialdehyde dehydrogenase	1.73	**-3.65**	-1.97
EJJ34_08570	*pyrK*	Dihydroorotate oxidase B electron transfer subunit	4.64	**-3.64**	—
EJJ34_08560	*pyrAA*	Carbamoyl-phosphate synthase pyrimidine-specific small chain	5.03	**-3.61**	1.38
EJJ34_10765	*yoyD*	DUF3311 domain-containing protein	3.14	**-3.59**	—
EJJ34_08575	*pyrD*	Dihydroorotate dehydrogenase	4.11	**-3.50**	—
EJJ34_05180	*cspB*	Cold-shock protein CspB	4.82	**-3.31**	1.46
EJJ34_08585	*pyrE*	Orotate phosphoribosyltransferase	4.41	**-3.31**	1.06
EJJ34_18065	*yvzF*	DUF3970 domain-containing protein	3.81	**-3.26**	—
EJJ34_22110	*yybK*	DUF2705 domain-containing protein	3.88	**-3.12**	—
EJJ34_20950	*dltX*	Teichoic acid D-Ala incorporation-associated protein DltX	3.33	**-3.04**	—
EJJ34_08555	*pyrC*	Dihydroorotase	4.93	**-3.02**	1.86
EJJ34_03485	*groES*	Co-chaperone GroES	2.82	**-3.02**	—
EJJ34_08030	*abh*	AbrB/MazE/SpoVT family DNA-binding domain-containing protein	2.61	**-2.98**	—
EJJ34_05480	*yheI*	Multidrug resistance ABC transporter ATP-binding/permease	3.50	**-2.94**	—
EJJ34_08120	*yktA*	UPF0223 family protein	3.31	**-2.92**	—

Supplementary Table S7: The most up-regulated genes comparing MB with WT

Gene ID	Gene name	Function	log2 (fold change)		
			TM vs WT	MB vs TM	**MB vs WT**
EJJ34_22760	-	Phage gp6-like head-tail connector protein	—	4.57	**4.23**
EJJ34_15785	*lrgA*	Antiholin-like protein LrgA	6.90	-2.65	**4.21**
EJJ34_15780	*lrgB*	Antiholin-like protein LrgB	5.95	-1.82	**4.10**
EJJ34_15490	-	tRNA-Arg	—	4.23	**3.90**
EJJ34_16735	-	Hypothetical protein	—	—	**3.88**
EJJ34_22560	-	Thioredoxin	—	4.19	**3.86**
EJJ34_11505	*yopB*	XRE family transcriptional regulator	3.79	—	**3.75**
EJJ34_11210	*yorH*	Hypothetical protein	2.39	1.15	**3.51**
EJJ34_05945	***yhjR***	Spore coat protein	-2.70	6.15	**3.40**
EJJ34_04555	***acoA***	acetoin:2%2C6-Dichlorophenolindophenol oxidoreductase subunit alpha	-7.38	10.82	**3.40**
EJJ34_22800	-	Phage terminase small subunit P27 family	—	3.73	**3.40**
EJJ34_00515	-	tRNA-Leu	2.74	—	**3.19**
EJJ34_11320	*yoqK*	Hypothetical protein	2.16	1.03	**3.13**
EJJ34_20750	*ywcE*	Spore morphogenesis/ germination protein YwcE	1.95	1.06	**2.99**
EJJ34_07065	*xkzB*	Hypothetical protein	1.24	1.73	**2.90**
EJJ34_08550	*pyrB*	Aspartate carbamoyltransferase	5.78	-2.84	**2.89**
EJJ34_16870	-	tRNA-Gly	3.25	—	**2.81**
EJJ34_11435	*yopN*	Hypothetical protein	3.16	—	**2.66**
EJJ34_21665	***yxbB***	Class I SAM-dependent methyltransferase	-1.97	4.66	**2.64**
EJJ34_02745	*cmpA*	Cortex morphogenetic protein CmpA	—	2.91	**2.59**

Supplementary Table S8: The most down-regulated genes comparing MB with WT

Gene ID	Gene name	Fuction	log_2 (fold change)		
			TM vs WT	MB vs TM	**MB vs WT**
EJJ34_18365	*ssrA*	Transfer-messenger RNA	-15.20	—	**-18.09**
EJJ34_06250	*argJ*	Bifunctional ornithine acetyltransferase/N-acetylglutamate synthase	-5.94	1.97	**-4.01**
EJJ34_06265	*carA*	Carbamoyl-phosphate synthase arginine-specific small chain	-7.71	3.85	**-3.90**
EJJ34_06245	*argC*	N-acetyl-gamma-glutamyl-phosphate reductase	-4.43	0.61	**-3.86**
EJJ34_08145	*yktD*	Class I SAM-dependent methyltransferase	-5.78	1.98	**-3.85**
EJJ34_08175	*ylaE*	Hypothetical protein	-4.29	—	**-3.76**
EJJ34_06255	*argB*	Acetylglutamate kinase	-6.25	2.53	**-3.75**
EJJ34_16065	*argG*	Argininosuccinate synthase	-5.51	1.91	**-3.65**
EJJ34_06260	*argD*	Acetylornithine transaminase	-5.97	2.43	**-3.59**
EJJ34_06270	*carB*	Carbamoyl-phosphate synthase arginine-specific large chain	-7.44	3.93	**-3.55**
EJJ34_05735	-	Hypothetical protein	-6.09	2.63	**-3.50**
EJJ34_22210	-	Tetracycline resistance efflux system leader peptide	—	-2.86	**-3.50**
EJJ34_09660	*cotU*	Hypothetical protein	-2.80	—	**-3.39**
EJJ34_17840	*sspG*	Acid-soluble spore protein SspG	-4.60	1.30	**-3.38**
EJJ34_19650	*cotG*	Spore coat protein G	-8.41	5.09	**-3.36**
EJJ34_16060	*argH*	Argininosuccinate lyase	-5.62	2.31	**-3.34**
EJJ34_17845	*yurS*	Hypothetical protein	-7.28	4.09	**-3.25**
EJJ34_02205	*yczN*	YjcZ family sporulation protein	-3.80	—	**-2.99**
EJJ34_14240	*yqbH*	DUF3599 family protein	—	-2.13	**-2.99**
EJJ34_09690	*cotC*	Spore coat protein CotC	-3.49	—	**-2.78**

Supplementary Table S9: The biofilm-related genes differentially expressed among strain WT, TM, and MB

Gene	Function/product	log$_2$ (fold change)		
		TM vs WT	MB vs TM	MB vs WT
**Transcriptional regulator**				
spo0A	Sporulation transcription factor Spo0A	—	—	—
spo0B	Sporulation protein	1.56	-1.39	—
kinA	PAS domain-containing protein	1.18	—	—
kinB	Two-component sensor histidine kinase	1.84	—	—
kinC	PAS domain-containing sensor histidine kinase	2.30	-1.64	—
kinD	Two-component sensor histidine kinase	—	—	—
kinE	Sporulation kinase	—	—	—
abh	AbrB/MazE/SpoVT family DNA-binding domain-containing protein	2.61	-2.98	—
abrB	Transition state regulatory protein AbrB	2.81	-1.34	1.43
sinI	DNA-binding anti-repressor SinI	2.50	-1.27	1.19
sinR	Transcriptional regulator	—	-1.42	—
sigH	RNA polymerase sigma-H factor	1.27	-1.18	—
ccpA	LacI family transcriptional regulator	—	—	—
veg	Hypothetical protein	1.18	-1.16	—
comER	Late competence protein	-4.36	4.14	—
slrA	DNA-binding anti-repressor SinI	2.48	-1.67	—
slrR	helix-turn-helix domain-containing protein	1.75	—	1.46
yvrH	DNA-binding response regulator	1.33	—	—
sigX	RNA polymerase sigma factor SigX	2.58	-1.77	—
degU	DNA-binding response regulator	—	—	—
degS	Signal transduction histidine kinase	—	—	—
**Extracellular matrix**				
tapA (*yqxM*)	TasA anchoring/assembly protein	1.52	—	1.23
sipW	Signal peptidase I	—	1.91	1.35
tasA	Spore coat protein	—	2.29	1.45
bslA(*yuaB*)	Biofilm surface layer	—	—	—
epsA	Hypothetical protein	1.89	-1.48	—
epsB	Tyrosine protein kinase	—	1.31	—
epsC	Polysaccharide biosynthesis protein	—	—	—
epsD	Glycosyl transferase	—	—	—
epsE	Glycosyl transferase	1.78	—	1.25

epsF	Glycosyl transferase	—	—	—
epsG	Membrane protein	—	—	—
epsH	Glycosyl transferase	—	—	—
epsI	Pyruvyl transferase	—	—	—
epsJ	Glycosyl transferase	—	—	—
epsK	Membrane protein	—	—	—
epsL	Sugar transferase	—	—	—
epsM	Acetyltransferase	—	—	—
epsN	Pyridoxal phosphate-dependent aminotransferase	—	—	—
epsO	Pyruvyl transferase	—	—	—

Supplementary Table S10: The sporulation-related genes differentially expressed among strain WT, TM, and MB

Gene	Function/product	log$_2$ fold change		
		TM vs WT	MB vs TM	MB vs WT
**Key transcriptional regulators during sporulation**				
sigA	RNA polymerase sigma factor SigA	—	—	—
sigH	RNA polymerase sporulation sigma factor SigH	1.26	-1.18	—
sigF	RNA polymerase sporulation sigma factor SigF	-2.34	2.22	—
sigE	RNA polymerase sporulation sigma factor SigE	-3.20	3.32	—
sigG	RNA polymerase sporulation sigma factor SigG	-1.61	1.87	—
spo0A	Sporulation transcription factor Spo0A	—	—	—
spo0B	Sporulation initiation phosphotransferase B	1.56	-1.39	—
spo0E	Aspartyl-phosphate phosphatase Spo0E family protein	1.29	—	1.10
spo0F	Sporulation initiation phosphotransferase Spo0F	—	—	—
spo0M	Sporulation control protein Spo0M	—	—	-1.67
rsfA	Prespore-specific transcriptional regulator RsfA	-6.61	6.51	—
spoIIID	Stage III sporulation protein D	-6.65	6.65	—
ylbO	RsfA family transcriptional regulator	-2.50	1.22	-1.33
spoVT	Stage V sporulation protein T	1.16	—	—
gerE	Spore germination protein GerE	-3.85	3.56	—
gerQ	Spore coat protein GerQ	-7.09	6.04	-1.09
gerT	Germination protein GerT	-5.12	6.23	1.07
**Stage II sporulation genes**				
spoIIAA	Anti-sigma F factor antagonist; Stage II sporulation protein AA	—	—	—
spoIIAB	Anti-sigma F factor	-1.98	2.00	—
spoIIB	Stage II sporulation protein B	-3.72	4.14	—
spoIID	Stage II sporulation protein D	-1.61	—	—
spoIIE	Stage II sporulation protein E	-2.45	2.34	—
spoIIGA	Stage II sporulation protein GA	-1.51	1.91	—
spoIIM	Stage II sporulation protein M	-3.53	3.08	—
spoIIP	Stage II sporulation protein P	-2.65	2.39	—
spoIIQ	Stage II sporulation protein Q	-7.25	7.26	—
spoIIR	Stage II sporulation protein R	1.15	—	—
**Stage III sporulation genes**				
spoIIIAA	Stage III sporulation protein AA	-7.13	6.84	—
spoIIIAB	Stage III sporulation protein spoab	-4.32	4.55	—
spoIIIAC	Stage III sporulation protein AC	-3.47	5.12	1.61

spoIIIAD	Stage III sporulation protein AD	-4.57	4.99	—
spoIIIAE	Stage III sporulation protein AE	-3.24	3.64	—
spoIIIAF	Stage III sporulation protein AF	-4.89	5.11	—
spoIIIAG	Stage III sporulation protein AG	-4.69	4.55	—
spoIIIAH	Stage III sporulation protein AH	-6.23	5.72	—
spoIIID	Sporulation transcriptional regulator	-6.65	6.65	—
**Stage IV sporulation genes**				
spoIVA	Stage IV sporulation protein A	-7.86	6.75	-1.14
spoIVB	Spoivb peptidase; stage IV sporulation protein B	-3.05	3.38	—
spoIVCA	recombinase family protein	-1.02	1.14	—
spoIVFA	Stage IV sporulation protein FA	-1.88	1.03	—
spoIVFB	Stage IV sporulation protein FB	-1.29	1.16	—
yqfD	Stage IV sporulation protein	-2.06	1.44	—
**Stage V sporulation genes**				
spoVAA	Stage V sporulation protein AA	-3.19	3.98	—
spoVAB	Stage V sporulation protein AB	-3.75	3.50	—
spoVAC	Stage V sporulation protein AC	-3.52	3.13	—
spoVAD	Stage V sporulation protein AD	-1.67	1.35	—
spoVAE1	Stage V sporulation protein AE	-3.22	2.68	—
spoVAE2	Stage V sporulation protein AE	—	—	—
spoVAF	Stage V sporulation protein AF	-2.36	2.16	—
spoVB	Stage V sporulation protein B	-2.86	2.16	—
spoVD	Stage V sporulation protein D	–	–	—
spoVE	Stage V sporulation protein E	-5.31	5.26	—
spoVFA	Dipicolinate synthase subunit A	2.20	-1.77	—
spoVG	Septation protein SpoVG	-2.61	3.18	—
spoVK	Stage V sporulation protein K	–	—	—
spoVM	Stage V sporulation protein M	-5.24	4.31	—
spoVR	Stage V sporulation protein SpoVR	–	–	—
spoVS	Stage V sporulation protein SpoVS	1.16	—	—
spoVT	Stage V sporulation protein T	-3.19	3.98	—
**Stage VI sporulation genes**				
spoVID	LysM peptidoglycan-binding domain-containing protein	-6.23	5.35	—
spoVIF	Sporulation-specific transcription factor	-7.31	6.20	-1.15

Supplementary Table S11: The expression of genes spontaneously mutated in the MB strain

Gene ID	Gene name	Fuction	$\log_2$ (fold change)		
			TM vs WT	MB vs TM	MB vs WT
EJJ34_18080	*gerAC*	Germination protein A3	—	1.74	—
EJJ34_15400	*hemA*	Glutamyl-tRNA reductase	—	—	—
EJJ34_09320	*mutS*	DNA mismatch repair protein MutS	—	-1.15	-1.01
EJJ34_12435	*trpC*	Indole-3-glycerol phosphate synthase TrpC	-3.12	2.94	—
EJJ34_18140	*yvrD*	SDR family oxidoreductase	—	—	—

Supplementary Table S12: The expression of genes related to secondary metabiotic products in strain WT, TM, and MB

Gene ID	Gene name	Fuction	log2 (fold change)		
			TM vs WT	MB vs TM	MB vs WT
EJJ34_01215	*skfA*	Sporulation killing factor	-5.87	5.82	—
EJJ34_01220	*skfB*	Sporulation killing factor system radical SAM maturase	-3.62	5.43	1.78
EJJ34_01225	*skfC*	CPBP family intramembrane metalloprotease	-1.56	2.37	—
EJJ34_11790	*sunT*	SPBc2 prophage-derived Sublancin-168-processing and transport ATP-binding protein SunT	3.62	-2.24	1.33
EJJ34_19115	*cypX*	Cytochrome P450%2C cyclodipeptide synthase-associated	-2.15	1.90	—
EJJ34_19120	*yvmC*	cyclo(L-leucyl-L-leucyl) synthase	-1.81	1.76	—
EJJ34_10050	*ppsA*	Non-ribosomal peptide synthetase	-2.17	1.20	-1.01
EJJ34_10045	*ppsB*	Plipastatin synthase subunit B	-2.47	1.55	—
EJJ34_10025	*ppsD*	Plipastatin synthase subunit D	-2.18	—	-1.31
EJJ34_10020	*ppsE*	Non-ribosomal peptide synthetase	-2.08	1.53	—
EJJ34_09355	*pksC*	Malonyl CoA-ACP transacylase	1.36	—	—
EJJ34_09360	*pksD*	Acyltransferase domain-containing protein	1.89	-1.14	—
EJJ34_09395	*pksJ*	Amino acid adenylation domain-containing protein	-1.54	—	—
EJJ34_09410	*pksN*	Amino acid adenylation domain-containing protein	-1.73	—	-1.13
EJJ34_09415	*pksR*	Methyltransferase	-2.34	—	-1.57

Reference

1. Shannon, P.; Markiel, A.; Ozier, O.; Baliga, N.S.; Wang, J.T.; Ramage, D.; Amin, N.; Schwikowski, B.; Ideker, T. Cytoscape: a software environment for integrated models of biomolecular interaction networks. *Genome Res*. **2003**, *13*, 2498-504.
